# Household air pollution in Nairobi's slums: A long-term policy evaluation using participatory system dynamics

**DOI:** 10.1016/j.scitotenv.2018.12.430

**Published:** 2019-04-10

**Authors:** K. Dianati, N. Zimmermann, J. Milner, K. Muindi, A. Ezeh, M. Chege, B. Mberu, C. Kyobutungi, H. Fletcher, P. Wilkinson, M. Davies

**Affiliations:** a University College London, UK; b London School of Hygiene and Tropical Medicine, UK; c African Population and Health Research Center, Kenya; d BuroHappold, UK

**Keywords:** Household air pollution, System dynamics, Group model building, Participatory modelling, Health impact assessment, Informal settlements, Kenya, Nairobi

## Abstract

58% of Nairobi's population live in informal settlements in extremely poor conditions. Household air pollution is one of the leading causes of premature death and disease in these settlements. Regulatory frameworks and government budgets for household air pollution do not exist and humanitarian organisations remain largely inattentive and inactive on this issue. The purpose of this paper is to evaluate the effectiveness of potential indoor-air related policies, as identified together with various stakeholders, in lowering household air pollution in Nairobi's slums. Applying a novel approach in this context, we used participatory system dynamics within a series of stakeholder workshops in Nairobi, to map and model the complex dynamics surrounding household air pollution and draw up possible policy options. Workshop participants included community members, local and national policy-makers, representatives from parastatals, NGOs and academics. Simulation modelling demonstrates that under business-as-usual, the current trend of slowly improving indoor air quality will soon come to a halt. If we aim to continue to substantially reduce household PM_2.5_ levels, a drastic acceleration in the uptake of clean stoves is needed. We identified the potentially high impact of redirecting investment towards household air quality monitoring and health impact assessment studies, therefore raising the public's and the government's awareness and concern about this issue and its health consequences. Such investments, due to their self-reinforcing nature, can entail high returns on investment, but are likely to give ‘worse-before-better’ results due to the time lags involved. We also discuss the usefulness of the participatory process within similar multi-stakeholder contexts. With important implications for such settings this work advances our understanding of the efficacy of high-level policy options for reducing household air pollution. It makes a case for the usefulness of participatory system dynamics for such complex, multi-stakeholder, environmental issues.

## Highlights

• HAP is an important health risk in low-income urban settings, such as Nairobi's slums.
• Under business-as-usual, the current trend of slowly improving indoor air quality will stop.
• For it to continue, a drastic acceleration in take-up of clean cookstoves is needed.
• This needs money, and one way to raise money is to invest in HIA and monitoring.
• This can turn a cycle of ‘non-attention and no funds’ into one of ‘raised awareness and more resources’.

## Graphical abstract

**Figure:**
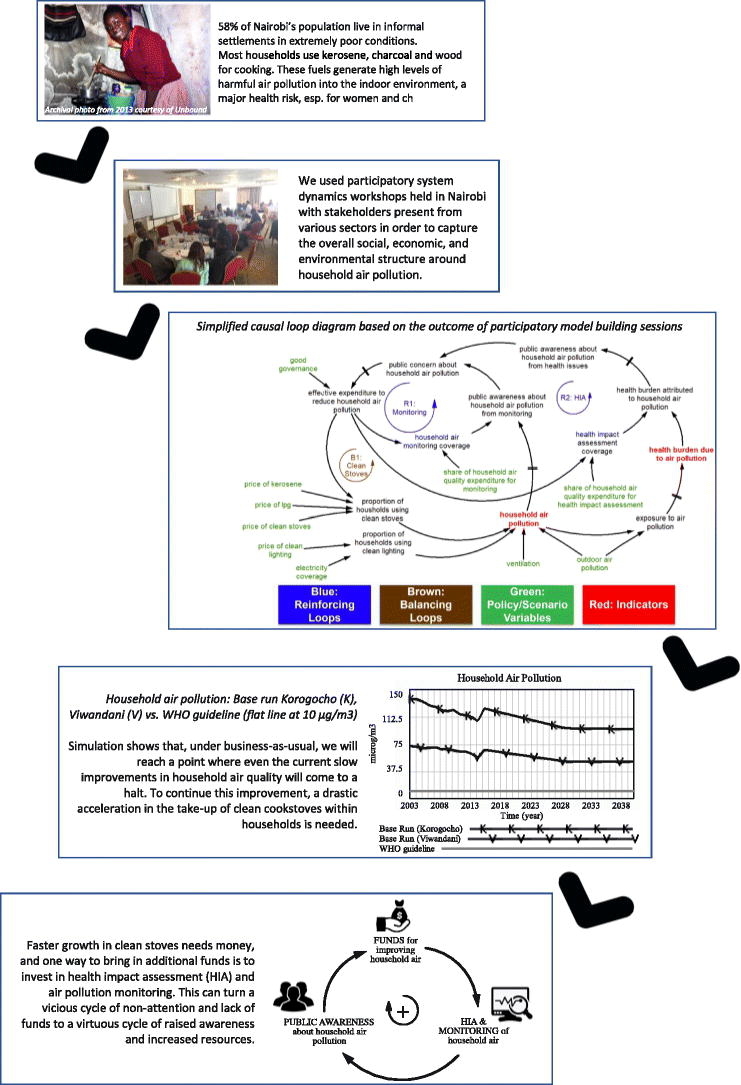

## 1 Introduction

Nairobi city, according to the most recent national census, is home to 3.14 million inhabitants (Kenya National Bureau of Statistics, 2010), having grown from just under half a million at the country's independence in 1963. The city's population growth is fuelled both by natural increase and migration from rural and other urban areas. For a long time, Nairobi has been the country's principal city and it remains an attractive destination for people looking for livelihood opportunities that are lacking in the mostly agricultural rural areas.

The rapid growth of the city's population has not been accompanied by sufficient provision of affordable housing and other social amenities, leading to the proliferation of slum settlements. It is estimated that Nairobi has over 150 slum settlements, scattered across the city. These settlements, which occupy <5% of the city's land mass, are home to an estimated 60–70% of the city population (Beguy et al., 2015). Numerous studies have reported the challenges that slum residents face, including the near absence of the public sector and poor access to public goods and services, with negative implications for various health outcomes (Kyobutungi et al., 2008; Mugisha, 2006).

Typical housing units in Nairobi's slums have tin/corrugated iron roofing and mud or tin/corrugated iron sheet walls. Most households rent one room measuring about 10 ft. by 10 ft. and these rooms serve as the kitchen, bedroom and living room (APHRC, n.d.). The rooms usually have one door and one window, although in some cases there are no windows at all. Most households rely on kerosene (paraffin) for cooking and lighting as well as charcoal or wood for cooking. In the poorest of households, the use of plastic waste, cloth rags and other unconventional fuels has been reported (Muindi et al., 2014). These fuels generate high levels of potentially harmful air pollution into the indoor environment. A separate study recorded high levels of particulate matter with aerodynamic diameter of 2.5 μm and less (PM_2.5_), especially in the evenings and in households burning charcoal/wood and kerosene (Muindi et al., 2016). In addition to housing features and behaviours that impact on the air quality, slums tend to be in areas close to primary sources of air pollutants. For example, many slums are built near busy highways, within industrial zones or near open dumpsites. Outdoor measurements of PM_2.5_ concentrations in slum areas found that there was spatial and temporal variations with slum villages close to major outdoor sources such as dumpsites having higher concentrations, while mornings and evenings were also noted to have elevated levels (Egondi et al., 2016). In a context of weak or non-existent policies to minimize emissions from various sources, slums experience high exposure to air pollution compared to non-slum areas of the city.

Household air pollution is estimated to result in a global burden on mortality of around 2.6 million premature deaths each year (GBD 2016 Mortality Collaborators, 2017). The most important contributor is biomass burning for cooking or heating, used by roughly half the world's population. The situation is exacerbated by factors including poor housing, inadequate ventilation and overcrowding, with women and children often exposed to particularly high levels of pollution. It has been established that large benefits can be gained from reducing air pollution, e.g. through switching to cleaner fuel sources such as LPG (liquid petroleum gas) (Morgan, 2015). Despite apparent benefits, in Kenya, to the best of the authors' knowledge, there are currently no programmes focusing on air pollution from a health point of view, either at national or at county level. This might be due to lack of specific internal or donor funding but could also be the result of a lack of understanding at governmental level on the magnitude and importance of the issue.

This paper therefore aims to investigate the comparative effects of a series of policy options on household air pollution in two of Nairobi's numerous slums, Korogocho and Viwandani. It analyses prerequisites, implications, and significance of public awareness and concern about household air pollution. We develop a system dynamics model that captures major drivers of household air pollution and potential policies aimed at mitigating them. We estimate potential improvements in the weight of the health burden associated with household air pollution as a result of various combinations of policies in order to identify beneficial policy directions.

## 2 Methods

This study combines methods including participatory modelling workshops, system dynamics (SD) modelling, and health impact assessment (HIA). In the following sub-sections, we introduce the study sites as well as each method, review the structure of the SD model, and validate the model to existing data.

### 2.1 The study sites: Korogocho and Viwandani

Korogocho is a slum settlement to the northeast of Nairobi city about 12 km from the city centre. It borders the Dandora dumpsite, Nairobi's official municipal dumpsite which is the final resting place for mixed waste streams from the city. This has been a source of pollution for residents of surrounding communities, with air pollution from burning garbage as well as soil and water pollution being key challenges. Viwandani is a slum settlement in the industrial zone of the city, with industries being visible sources of air and water pollution in the area. It lies about seven kilometres from the city centre and is home to a youthful, more educated and highly mobile population seeking employment in industries. In contrast Korogocho's population is older and less educated, and the majority have lived in the slum longer, compared with Viwandani's residents. Both slums face a shared challenge of poverty and exclusion especially with regards to the provision of government services such as health and education (Emina et al., 2011).

### 2.2 Participatory system dynamics workshops

The issue of household air pollution concerns various stakeholders besides the residents, including local decision makers, government policymakers, parastatals, and non-governmental actors. Therefore, to increase the chances of research resulting in increased awareness and commitment to change among all actors involved, we organised a series of three multi-stakeholder workshops in Nairobi and used participatory system dynamics to frame the discussions. Participants included a diverse group of stakeholders, including individuals with expertise on air quality and its impacts on health, as well as those working on policy development and implementation. Attendants also included community members from both slums, academia, representatives from the local and national governments, national parastatals, national and international NGOs, and United Nations agencies.

In our modelling session, we asked participants to identify the most central variables concerning indoor air quality. These variables were then gradually added to a causal loop diagram on a large whiteboard by asking participants to identify the chains of causality within the system. Following this process, we captured the overall causal structure of the system within the model and identified any emerging feedback loops, i.e. chains of causal relationships involving circular causality.

Each workshop was followed by off-site refinement, formalisation and calibration of the system dynamics model, where the model was further elaborated, adding more variables and closing some of the previously open feedback loops. As part of this refinement, we sent out a questionnaire to ten members of the stakeholder group asking them to rate various policies identified during the workshops based on their relative importance to household air pollution. Subsequently, out of a total of fourteen policies, we picked seven which were considered to be the most important, to be included in the model.

During the second and third workshop rounds, we sought to verify with the participants whether the model's components, inter-linkages, and resulting behaviour resonated with them and reflected their understanding of the many inter-related issues around indoor air pollution in Nairobi's slums.

‘*Participatory system dynamics modelling*’ and ‘*group model building*’ (Vennix, 1996) (terms which are sometimes used synonymously) are useful for organizing the collective knowledge of stakeholders in a visual structure that promotes learning and allows for constructive, targeted discussions. When tackling complex problems with multiple stakeholder groups involved, taking a participatory approach is preferred to ‘expert mode’, where the modellers construct a model ‘at their desk’ based on available sources of information (Antunes et al., 2015; Vennix, 1996). This combined approach allowed us to make use of the diverse set of expertise available in our interdisciplinary stakeholder groups, while complementing that with rigorous quantitative modelling to simulate the implications of the group's assumptions about the structure of the system. Allowing policymakers to rely on their own thinking process in collaboratively building a model engenders a sense of ownership and commitment to the outcome of the modelling process and in this way increases the chances of successful implementation of resulting policies (Vennix, 1996).

During our field trips, the project team also held two separate focus group discussions with community members from Korogocho and Viwandani, within the informal settlements where they live. The focus group discussions revolved around indoor air quality, barriers in the community's adoption of clean cook stoves and other issues touching on housing, outdoor air and community/individual agency to agitate for action against known polluters.

### 2.3 Health impact assessment

We used a life table model (Miller and Hurley, 2003) to quantify the impact of changes in exposure to household air pollution. The model was driven by changes in long-term (annual average) exposure to PM_2.5_, a key constituent of household air pollution and the most consistent and robust predictor of mortality due to air pollution in studies of long-term exposure (Cohen et al., 2017). Based on changes in PM_2.5_ exposure (generated by the system dynamics model), the life table model calculates changes in the pattern of deaths in the population over time and the corresponding impact of these on the duration of life, expressed as total life years gained or lost in the population. As seen later in the Scenario analysis section, this will enable us to observe how results of our policy scenarios in terms of differences in household air pollution translate to health outcomes in terms of the avoided life years lost. The baseline population and mortality data for the local population, used to set up the model, were obtained from the Nairobi Urban Health and Demographic Surveillance System (NUHDSS) (Emina et al., 2011).

### 2.4 Quantitative system dynamics

The qualitative causal diagram resulting from the participatory workshops was refined, and the health impacts were incorporated into the system dynamics model. We also wanted to understand the results of different policies on this complex model. Since such interactions are too large and complex to simulate mentally, computer simulation is the only practical way to test them (Richardson, 1986; Sterman, 2000). The inherent complexity observed in the structure of the system under investigation in this study makes SD modelling a highly suited methodology to deal with this complexity. It enhances our understanding of complex systems through transparent modelling of the systems' structure. Using computer simulation models, SD helps us pinpoint the sources of policy resistance, and thus, design more effective policies (Sterman, 2000). Therefore, we brought in real world data to develop a quantified and formal system dynamics simulation model from the collaborative maps generated through stakeholder workshops. We quantified and parameterised it before applying it for policy analysis.

#### 2.4.1 Model structure

The participatory process and subsequent off-site refinement resulted in a model with >150 variables. The model structure is identical for both slums, except that it is differently parametrised for each context.^1^ Due to its complexity, here we describe only a highly simplified causal loop diagram. The full model documentation is detailed in Appendix A. The model in its digital format, along with all scenario and sensitivity runs, can be found as Supplementary material published with this paper.

**Fig. 1:**
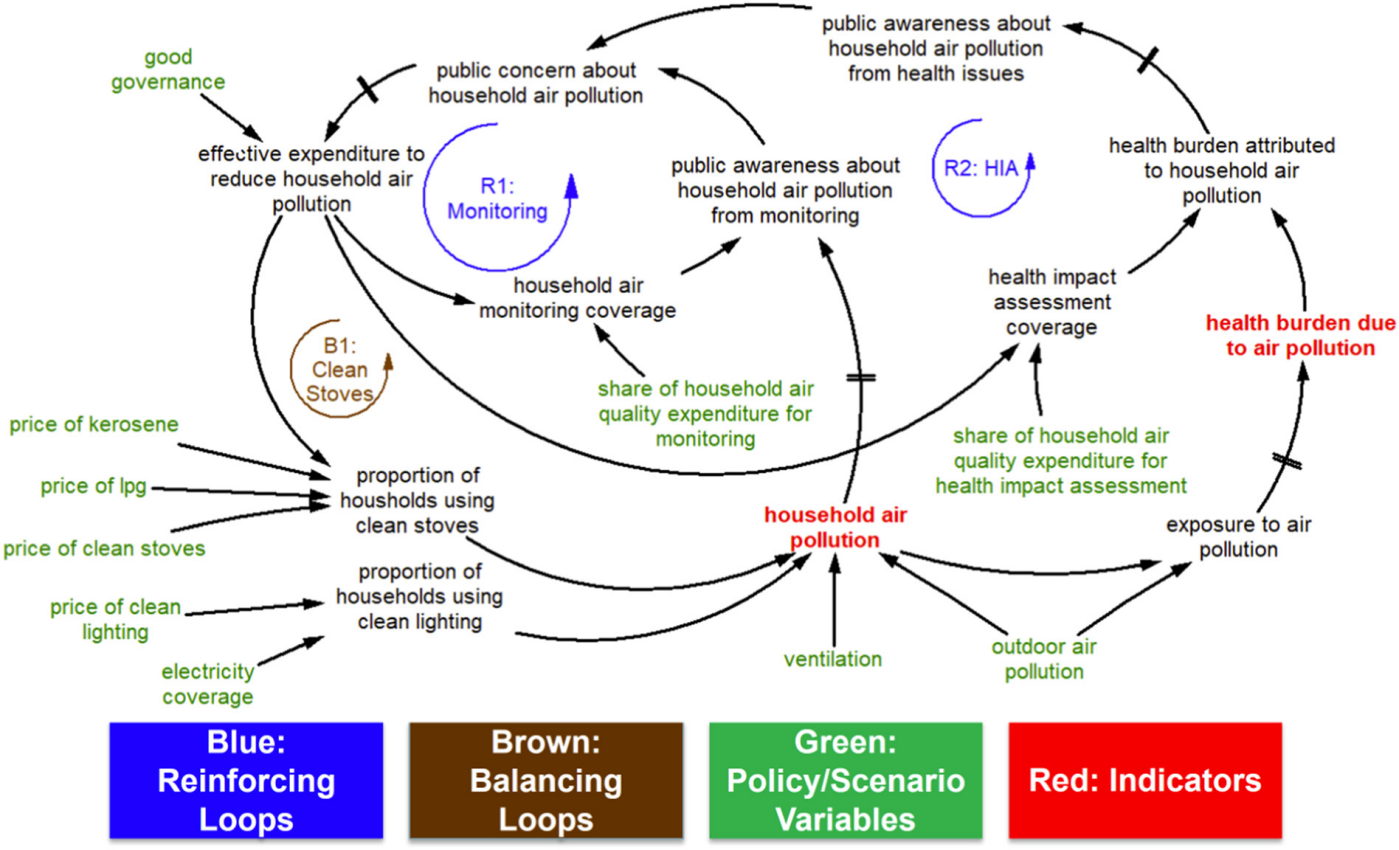
Simplified causal loop diagram of the model; hash marks on certain relationships represent delays.

Fig. 1 portrays the simplified causal loop diagram arrived at by distilling the key feedback structure of the formal system dynamics model. The legend explains the colour coding. Starting with the central variable of *household air pollution* highlighted in red as our main indicator, we will explain backwards (against the direction of the arrows) along the chains of causality to investigate the key dynamics of the system as modelled here. The key drivers of the average level of *household air pollution* (proxied in this study by the concentration of PM_2.5_) in Nairobi's slums are the levels of *outdoor air pollution* and *ventilation*, as external factors, and the *proportion of households using clean stoves*/*lighting* internally. This study focuses mainly on exploring the internal factors, i.e. the prevalence of clean appliances. In line with the findings of the workshops, we assume in our model that prevalence of clean lighting is mainly driven by the electricity grid coverage and to some extent by the prices of electric lights. The prevalence of clean cook stoves, on the other hand, is driven by their prices, relative prices of clean versus “dirty” fuels, and finally the levels of public expenditure in providing subsidised appliances. The lower the prices of clean cook stoves and/or clean fuels, the higher the take-up and usage of these by residents of the informal settlements.

The funds available for subsidising clean cook stoves come from the total funds spent for combatting household air pollution, *effective expenditure to reduce household air pollution*. This expenditure is modelled to be driven not only by *public concern about household air pollution*, but also by the extent of enforcement, political will and *good governance*. To capture this, we have used the World Bank's Worldwide Governance Indicators for Kenya (Kaufmann et al., 2010). This data consists of six separate indicators capturing various aspects of governance: (i) voice and accountability, (ii) political stability and absence of violence, (iii) government effectiveness, (iv) regulatory quality, (v) rule of law, and (vi) control of corruption. These are indicators ranging from −2.5 (weak) to (2.5) strong. We have averaged the six indicators and converted the result to an index between zero and one to come up with an aggregate past *governance indicator* (0.383 out of 1). The future target is set by the user as a policy/scenario variable (in green). While we acknowledge the fact that the situation in the two slums in this study differs from Kenya as a whole, nonetheless this data was the best proxy available for our purpose.

*Public concern about household air pollution* comes from two sources in the model: either direct monitoring of indoor air pollution levels or through awareness about the health burden associated with household air pollution, which can be estimated through HIA studies. The extent to which either monitoring or HIA are systematically carried out in the informal settlements depends on the levels of funds available for each, which are in turn determined by multiplying the total *effective expenditure to reduce household air pollution* by the share of this expenditure going to either of these initiatives. The extent of awareness and concern generated through each channel are also driven by the actual levels of indoor air pollution; directly so in the case of monitoring and in the case of health impact assessment, travelling through *exposure to air pollution*, *health burden due to air pollution* and *health burden attributed to household air pollution*. Exposure itself is a consequence of either *household* or *outdoor air pollution*. *Outdoor air pollution* lies outside the scope of this study (see Section 4.2) and it is fed as exogenous data to the model. Past levels of PM_2.5_, 166 μg/m^3^ for Korogocho and 67 μg/m^3^ for Viwandani, are set according to the limited available real-world data (APHRC, n.d.), and its future levels are incorporated as a scenario variable.

#### 2.4.2 Main feedback loops

The causal structure of the system dynamics model (Fig. 1) shows three noteworthy feedback loops within the system that might drive or counter change in the real world. *R1*: *Monitoring* and *R2*: *HIA* belong to the class of feedback loops known as ‘reinforcing’, while *B1*: *Clean Stoves* is known as a ‘balancing’ loop. The inherently different nature of these feedback loops can have a decisive effect on the ultimate success of policies.

In the *B1*: *Clean Stoves* balancing feedback loop, a potential increase in expenditure for clean cook stoves should, ceteris paribus, help bring down *household air pollution*. A decrease in air pollution is likely to make the public slightly less concerned about this issue, and a less worrisome public (be it the government, the communities, or NGOs) would then perhaps think that the issue has to some extent been contained and no longer warrants the previously increased level of allocated funds and decide to divert those additional funds to other more pressing problems, bringing the level of expenditure back close to the initial lower level; hence the use of the label ‘balancing’.

Yet, if expenditure for monitoring or health impact assessment studies is increased, once the results of such studies are published, this new information could make the public more anxious about indoor air pollution, which in the model leads to a higher budget allocated to this issue for the next year. Therefore, an increase in the *share of household air quality for monitoring*/*health impact assessment* has the potential to increase the available resources the next time round. This argument makes a theoretical case for allotting a share of the available budget to monitoring and health impact assessment, a policy that we are going to test in Section 3.2.2.

#### 2.4.3 Model validation

In SD modelling, validation depends on the model purpose, and it consists of an iterative process of building confidence in the usefulness of the model, rather than establishing its objective ‘truth’ (Barlas and Carpenter, 1990). There exist a number of well-established tests to help to verify the usefulness of the model to its purpose, which include both structural and behavioural tests (Barlas, 1996; Senge and Forrester, 1980). Our model has undergone several validity tests, both structurally and behaviourally, including dimensional consistency checks, extreme condition tests, behaviour-reproduction tests and behaviour-sensitivity tests. The structure has also been validated against expert opinion during the multi-stakeholder workshops as well as ongoing collaboration with co-authors from APHRC who have knowledge of the local context. The model has been parametrised using the limited numerical data available from various sources, including the NUHDSS database. Detailed information on model formulations can be found in Appendix A, and a full list of model parameters for the two contexts is reported in Appendix D. Selected sensitivity tests are reported in Appendix B.

**Fig. 2:**
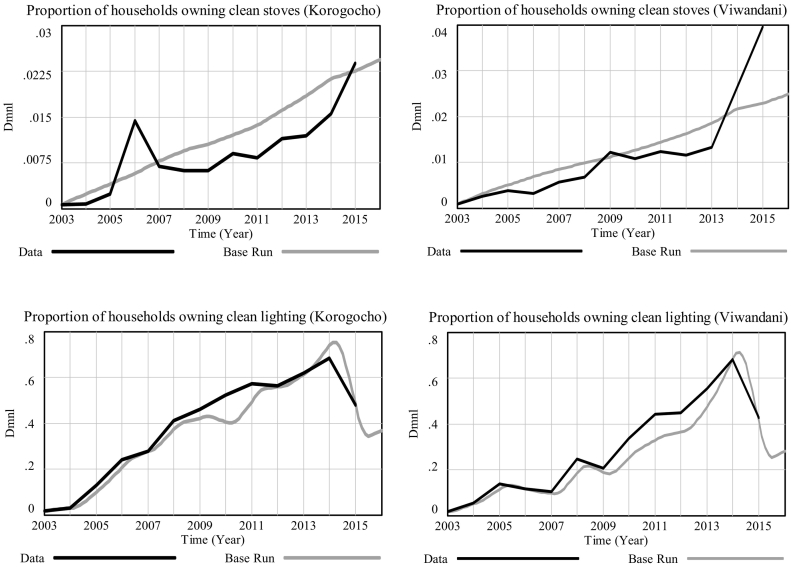
Proportion of households owning clean stoves (upper plots) and clean lighting (lower plots); black curve: historical data; grey curve: base simulation.

The behaviour of the model has also been validated against time-series data from the NUHDSS database. We start the model in 2003, where NUHDSS starts, and validate it using data available until today. For instance, in Fig. 2, the model's *Base Run* (grey curves) captures the general long-term trend in historical data (black curves) fairly well. Since the focus of this project is long term policymaking, the fact that short-term oscillations are not captured is not considered a limitation of the model for our purpose.

The prevalence of both clean stoves and clean lighting in the two slums under study has been generally increasing since 2003. Note, however, that the scales of the graphs are very different for clean stoves (upper plots) and clean lighting (lower plots), with the take up of clean stoves being far slower than that of clean lighting. The generally upward trend is only broken by the most recent datapoint for access to clean lighting in both communities. This fall could have been due to recent efforts to clamp down on illegal and unsafe connections especially widespread in slum areas. The fall is captured well by model simulation, as prevalence in clean lighting is tightly driven by access to electricity in our model, for which historical data is available. The idiosyncrasies of developments in ownership of clean stoves among households are however less straightforward, and the model only manages to capture the general upwards trend, mainly a result of a slow increase in funds available for the provision of clean cookstoves. In particular, the steep increase in the prevalence of clean stoves in both slums during 2014–2015 is thought to be due to a project called *Prima Gas*, which made LPG more affordable for low-income groups by allowing customers to partially refill their cylinders from a mobile refill point for the amount of cash they have in hand; starting at a minimum of 50 Kenyan shillings (CapitalFM, 2012). As this external driver is not accounted for in the model, we cannot replicate this recent steep rise. Note however that, as portrayed further ahead in Fig. 4, the scales of the curves for lighting and for stoves are so different that, when shown in the same graph, model simulation seems to overlap completely with historical data. As the same model structure with different parameters is used for both contexts, we maintain that the model has successfully passed the ‘family-member’ test as it can be said to represent a ‘family’ of social systems, i.e. the socio-physical system surrounding household air pollution in low-income slum settings.

The scarcity of available time-series data for important variables in the model, including our central indicator *household air pollution*, posed a challenge to the behavioural validation of the model. This is a limitation that entails a degree of caution regarding the use of the model as the only input to policymaking. Nevertheless, while taking such limitations into account, the model still offers valuable insights for policy, as we will further report in the next sections.

## 3 Results

In this section, we will start by examining the *Base Run*, which is the model's projection of current trends under business-as-usual. Next, we will explore three different scenarios and consider potential implications.

### 3.1 Base run

Firstly, let us look at future developments of our main indicators according to the model's projections of current trends under business-as-usual. These projections are not merely extrapolations of current trends. Instead, variables can undergo changes in trend, as the behaviour of the model is driven by its structure, and not by its inputs. For brevity, some of the scenario analysis graphs are presented only for Korogocho. In these instances, the results for Viwandani show similar behaviour with identical implications.

**Fig. 3:**
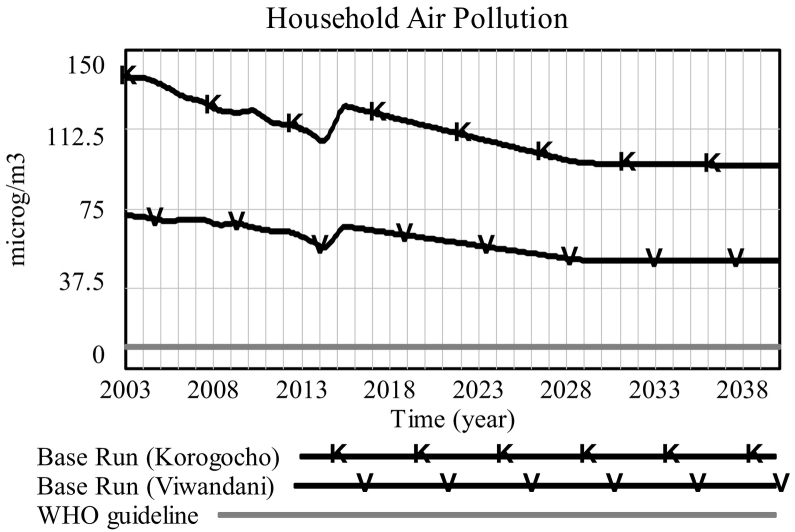
Household air pollution: *Base run Korogocho* [*K*], *Viwandani* [*V*] vs. *WHO guideline* (*flat line at* 10 μg/m^3^).

**Fig. 4:**
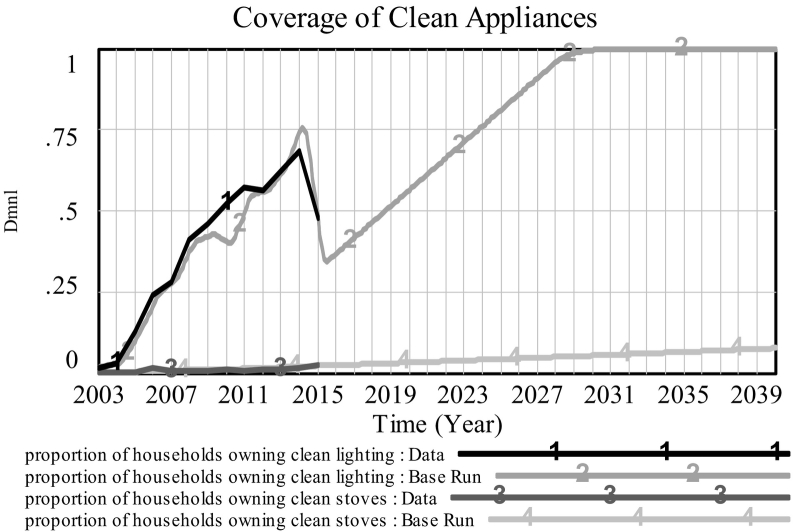
[Korogocho] Coverage of clean lighting ([1] Data, [2] Base Run), and clean stoves ([3] Data, [4] Base Run).

Allowing the model to run up to 2040 (Fig. 3), we see that *household air pollution* (proxied by PM_2.5_ concentration) continues to fall slowly (in both slums), before reaching a plateau around 2030. The available data provides reasonable historical evidence on the prevalence of clean appliances since 2003, which enables us to postulate the implied behaviour of *household air pollution* from our model. This suggests that *household air pollution* has been slowly falling over recent years, indicating a gradual improvement in household air quality, which is a result of electric lights replacing polluting kerosene lights in most households. An exception to this generally improving trend occurs in the year 2014, when prevalence of electric lighting actually *falls* (Fig. 4), as mentioned earlier in Model validation, and *household air pollution* consequently rises. According to the simulation, we will eventually reach a point where almost all households in the informal settlements under study have access to electricity and electric clean lighting.^2^ Therefore, from that point onwards the only way, within the boundaries of our model, to further reduce the generation of air pollution in households is through more extensive take-up of clean cook stoves. However, as depicted in Fig. 4 (Korogocho only), growth in the prevalence of clean stoves is completely dwarfed by that of clean lighting. In other words, the former is so slow that the resulting improvements from clean stoves are almost imperceptible once the prevalence of clean lighting reaches saturation around 2030. Therefore, under business-as-usual, we will reach a point where even the current slow improvements in household air quality will come to a halt. We will therefore explore some policy scenarios for achieving more substantial reductions in household air pollution.

### 3.2 Scenario analysis

#### 3.2.1 Description of scenarios

Policy and scenario variables used in the model are of three distinct types. The first type consists of what-if scenario variables concerning the prices of different fuel types, prices of stoves, and quality of governance. These are variables determined at a higher, usually national, administrative level. Secondly, there are those decisions that could be made at a local community level concerning the allocation of any available funds to spend towards mitigating household air pollution. It is assumed that these funds could be divided between direct provision of subsidised clean cookstoves to slum residents as well as indoor air monitoring and HIA initiatives. Finally, there are factors related with outdoor air pollution and ventilation, which are only crudely included in the model as exogenous drivers of household air pollution. These variables can be adjusted by the model user to observe effects of changes in outdoor air pollution and ventilation on closing in towards acceptable household air pollution levels.

We envisaged three scenarios corresponding to the three distinct types of policy and scenario variables described above. Our three scenarios are summarised in Table 1. The scenarios are additive. Our first scenario involves manipulating the prices of fuels and appliances. The second scenario adds a modified allocation of resources to that, so that more resources go towards monitoring and HIA. Finally, the third scenario adds an assumption of a substantial improvement in outdoor air quality and ventilation. A detailed characterization of these scenarios with regards to parameter values in the model can be found in Appendix C.

**Table 1:** Summarised description of scenarios.

Scenario	Summarised description	Notes
Scenario I: fuel and stove prices	• Lower LPG prices • Lower prices of clean stoves • Higher kerosene prices • Better governance	Adjusting prices of fuels can be attained by lowering/increasing subsidies. Lower stove prices could be a result of supporting local manufacturers. Funds for increasing LPG subsidies or supporting stove manufacturers can be sourced from savings on kerosene subsidies.
Scenario II: + monitoring and HIA	• All of the above, • Plus a higher share of available budget spent for monitoring and health impact assessment.	
Scenario III: + outdoor and ventilation	• All of the above, • Plus a drastic fall in outdoor air pollution, • Plus an improvement in ventilation (only for Korogocho)	This is the most comprehensive scenario.

Scenario I (*fuel and stove prices*) involves a redirection of subsidies from kerosene to LPG and to supporting local manufacturers of clean stoves. It also entails drastically improving the enforcement of any existing regulations, as well as reducing existing corruption that could lead to misallocations of available funds for tackling household air pollution. In the model, these assumptions are proxied by a step-wise 50% increase in kerosene prices, 25% decrease in prices of LPG, and 50% decrease in prices of clean cook stoves and clean lighting. These changes are assumed to be implemented in three steps: the first one in 2017, and then every three years in 2020 and 2023. We also assume a gradual 50% improvement in *good governance* by the end of our simulation period: 2040.

In *Scenario II* (+ *monitoring and HIA*), we accompany the above changes in policy with gradually ratcheting up the share of the available budget going towards monitoring and health impact assessment, up to 15% for each by 2023. This will gradually bring down the share of the available budget going to the provision and/or subsidising of clean cook stoves to 70% by 2023. It is worth noting that the size of the available budget is not fixed and is endogenously determined under the influence of *public concern about household air pollution*. The *effective* amount of funding is also mediated by *good governance*.

Finally, in our most comprehensive scenario, *Scenario III* (+ *outdoor and ventilation*), we complement the above indoor-air related policies with a drastic (50%) reduction in outdoor air pollution, and, in the case of Korogocho, a drastic (50%) improvement in ventilation, to demonstrate the potential of improving household air via improvements in outdoor air. In Viwandani, our base assumption for the degree of ventilation in households, estimated based on the limited data available, is already quite high (67%). Note that improvement in ventilation in the absence of improvements in outdoor air quality, can lead to a worsening of indoor air quality, as the outdoor is often more polluted than the indoor in Nairobi's slums.

#### 3.2.2 Results of scenarios

**Fig. 5:**
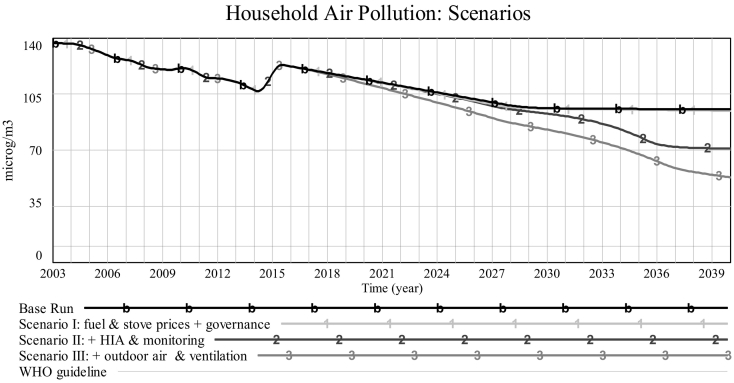
[*Korogocho only*] *Comparing* household air pollution *under different scenarios*.

We will now compare the results of these scenarios against each other and against the *Base Run*. In Fig. 5 (Korogocho only), the projected future path of *household air pollution* under various assumptions is shown. The graph shows how each scenario performs better than the previous one, thanks to a more comprehensive package of policies implemented. However, the scale of improvements resulting from adding each set of policies is different from other ones.

**Fig. 6:**
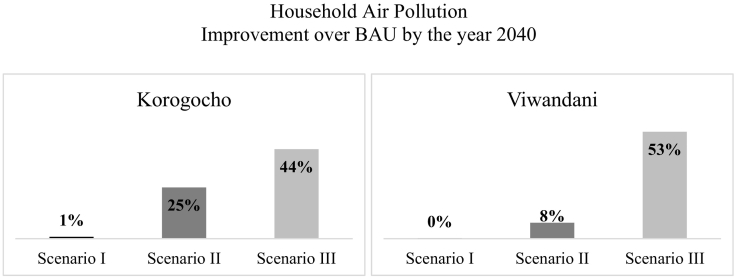
Household air pollution *under different scenarios by 2040*.

**Fig. 7:**
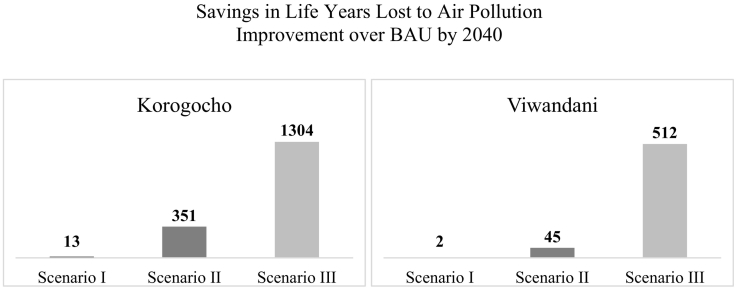
*Savings in* life years lost to air pollution *under different scenarios by 2040*. *Approx*. *population size*: *Korogocho 32*,*000*, *Viwandani 57*,*000*.

Fig. 6 gives a clearer picture of how the three scenarios fare against each other. This bar graph captures the improvement that each portfolio of policies generates above business-as-usual (*Base Run*), by 2040. This improvement in *household air pollution* results in a comparable improvement in *life years lost to air pollution*, as seen in Fig. 7. Results show that manipulating fuel subsidies and appliance prices alone, even combined with drastic improvements in *enforcement* (*Scenario I*) does not result in any substantial improvements by the end of our simulation period. If, however, we complement this by investing in monitoring and health impact assessment (*Scenario II*), we can hope for a much more significant betterment of indoor air quality that is likely to result in roughly proportionate improvements in *life years lost to air pollution* (Fig. 7). The best results by far, however, are only made possible via combining the above policies with a drastic reduction in outdoor air pollution and an improvement in ventilation (*Scenario III*).

Therefore, as projected by the model, policies like redirecting fuel subsidies to cleaner fuels, reducing stove prices, and strengthening *good governance*, even when combined, do relatively little to improve household air pollution. This is because currently the amount of funds allocated to combatting household air pollution is so low that incremental increases or reallocations fall short of achieving any substantial improvements over business-as-usual. This means that to see more visible impact, we require available funds to grow by orders of magnitude, which implies that public concern over household air pollution needs to be raised exponentially. This is precisely what investing in monitoring and health impact assessment can achieve thanks to the self-reinforcing nature of the feedback loops involved, as seen in Fig. 1.

Furthermore, what is striking is that even with investments in monitoring and health impact assessment (*Scenario II*), we would still end up far above the WHO guideline for acceptable exposure to annual average PM_2.5_ (10 μg/m^3^, thin black line in Fig. 5). This points to the fact that without tackling the sources of outdoor air pollution, it will not be possible to get indoor air pollution closer to acceptable levels. However, the alarming result from simulation is that even with *Scenario III* assumptions, that give the best results among our scenarios, we end up at a level of pollution that is still about five times higher than WHO guideline.

##### 3.2.2.1 Synergies among policies

**Fig. 8:**
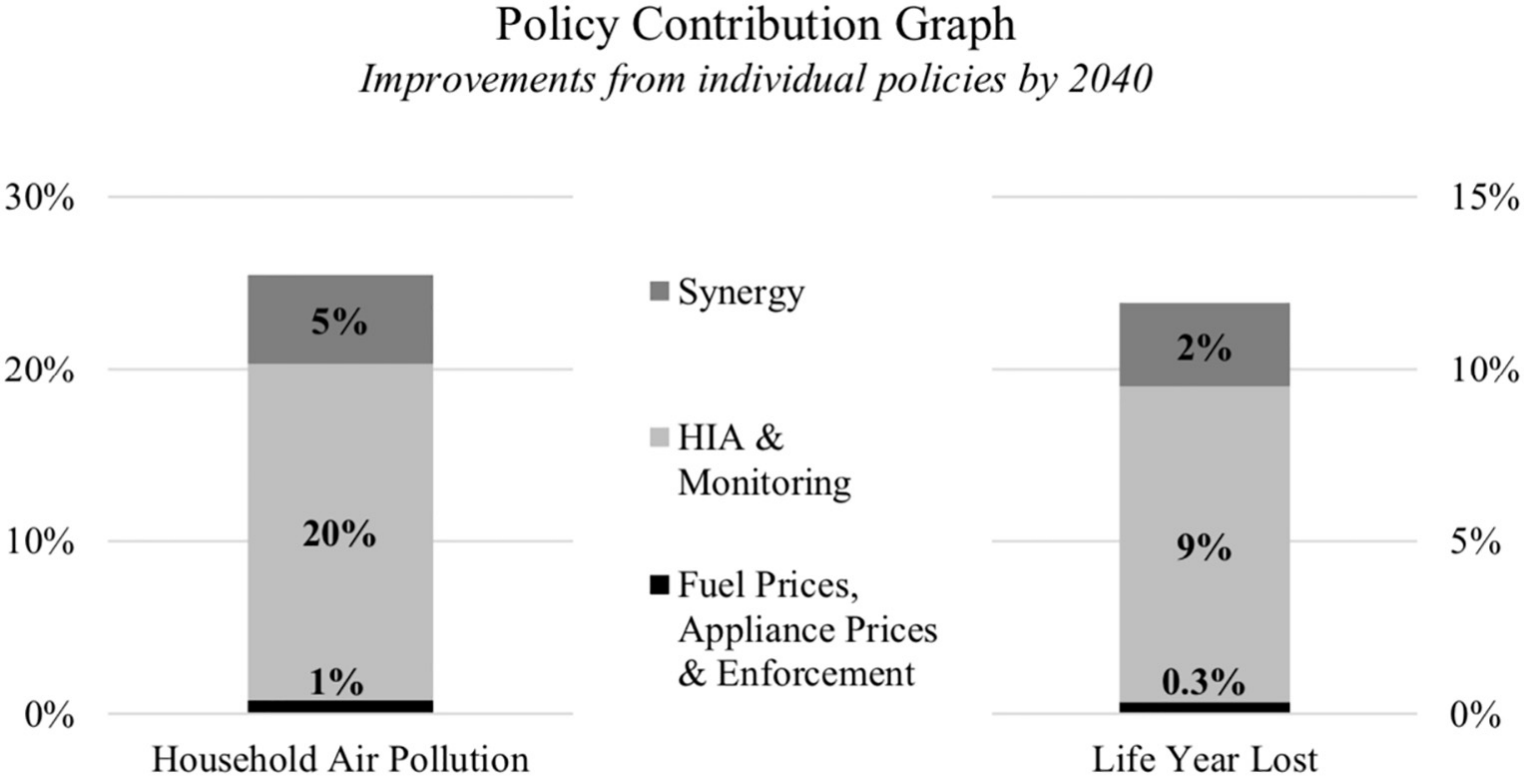
[Korogocho only] Individual policy contribution graph.

We saw earlier that our comprehensive portfolio of exclusively indoor-related policies, *Scenario II*, yields a 25.5% improvement in *household air pollution* and prevention of 351 potential life years lost to air pollution per year in Korogocho by 2040. But what is the contribution of each single policy in this total progress? Fig. 8 outlines these contributions for our two indicators of interest, *household air pollution* and *life years lost*. These values are obtained by simulating each individual policy separately, in the absence of any other interventions, and then comparing improvements against the baseline scenario.

From Fig. 8, it becomes clear that most improvements stem from investment in HIA and monitoring policies, which then helps bring in further funds for the provision of clean cookstoves. More interestingly, the substantial upper section of the contributions is not brought about by any individual policy, but from the *synergy* among all those implemented. These synergies are triggered only as a result of the *R1* and *R2* reinforcing feedback loops described in Section 2.4.2, and therefore depend on the implementation of HIA and monitoring. These reinforcing mechanisms can potentially enlarge the size of the ‘pie’ of available funding and make the resulting improvements larger than the sum of improvements from implementing single policies.

## 4 Discussion and conclusion

### 4.1 Findings and implications

In this study we built a quantitative system dynamics model on the problem of household air pollution in Nairobi's slums, using inputs obtained during rounds of multi-stakeholder participatory modelling workshops. We used the formal and tested system dynamics model to compare three hypothetical scenarios involving different portfolios of policies, which helped us in better understanding the dynamics of the socio-physical system.

Although there was not an abundance of numerical data for parameterisation, calibration and validation of the quantitative system dynamics model, which makes the model more suited to exploratory purposes, several well-founded and useful insights still emerged as a result of this study. Our results show that under business-as-usual, the current trend of slowly improving indoor air quality would come to a halt due to the saturation of the take-up of electric lighting and the extremely slow rate of take-up of clean stoves. This should be taken as a warning sign that if we aim to reach WHO's suggested guideline in terms of acceptable PM_2.5_ levels, a drastic acceleration in the take-up of clean stoves will be needed. According to our model's projections – without investing unfounded faith in their point-accuracy – even with a comprehensive package of indoor-air focused policies, there is little hope of closing the gap between status quo and WHO guidelines for air pollution by 2040.^3^ Even for the current downward trend to continue, our results, as well as our engagement with the community, have led us to believe that arriving anywhere near the WHO guideline will require addressing sources of outdoor air pollution, such as neighbouring dumpsites, industrial sites, traffic, etc. in parallel to sources of indoor air pollution. This will pose complications in implementation, as these dumpsites are sources of employment and livelihood for many slum residents.

Our simulation results also point to the potentially high impact of working towards raising the public's and the government's awareness and concern about household air pollution and its consequences for residents' health. To achieve this, our study suggests diverting some of the available budget (however big or small it is) to household air quality monitoring and health impact assessment studies, to ‘close the loop’ and bring the issue of household air quality higher up on the list of public/government priorities. Such investments, due to the self-reinforcing nature of the dynamics involved, can entail high return on investment, as the policymaker would be able to leverage the results of such studies to enlarge ‘the size of the pie’ of available money and resources (loops *R1* and *R2* in Fig. 1). We saw in the previous section how investments in monitoring and HIA have the potential to create synergies among existing policies. However, one must recognise that redirecting investments towards monitoring and health impact assessments may lead to slightly worse results in the short-term due to the time it takes before these policies pay off. In the world of politics, this delay may pose a serious implementation challenge.

The workshops held during this study engaged stakeholders in the gradual but rigorous process of developing a system dynamics model. It also demystified the completed model as stakeholders were involved in the model-building process from identifying simple relationships to complex inter-linkages of sectors. Testimonials from participants led us to believe that they found the process useful, both in terms of discovering aspects and dynamics of the air pollution issue which they were previously unaware of thanks to the expertise brought in by other stakeholders, and in terms of becoming familiar with ‘group model building’ as a powerful problem structuring and policy analysis method.

Previous studies have empirically assessed air pollution (Egondi et al., 2016; Muindi et al., 2016) and its impact on health (GBD 2016 Mortality Collaborators, 2017), or they have focused on the feeling of helplessness of slum residents towards the issue (Muindi et al., 2014). This study offers a framework to try and bring these diverse strands of research together. Through this work, we have contributed to the literature by addressing the issue of the slow take-up of clean cookstoves in low-income slum settings by bringing the physical, social and policy aspects together in an integrated quantitative model with a holistic and dynamic lens. We did this through engaging community members and local policymakers in the process with the aim of raising the issue's priority on their agendas and fostering a shared appreciation of important feedback mechanisms.

### 4.2 Limitations and future work

It is important to note that the model presented in this paper is derived through a participatory process with a particular set of stakeholders and therefore represents one possible model of the system that does not capture every possible mechanism. Therefore, we must stress that the model is not presented here as the definitive model of household air pollution in slums, but only as a highly simplified perspective that we believe is useful for deriving the sort of insights highlighted in the previous section.

One must also recognise the limitations imposed on this study due to a shortage of available time-series data on such key variables such as household and outdoor air pollution (where we had only one data point for each variable), as well as past expenditures on related policies, among other variables. It is not that such data externally drives the model, rather that such data would have been useful for a more in-depth comparison of simulated behaviour against real-world observations.

Importantly, while our replication of historical data for the *number of households owning clean lighting* was very good, the main driver of this indicator in our model is *electricity coverage*, which is taken as exogenous real-world data fed into the model, with an assumption of full electricity coverage of slums by 2040. In this respect, all our scenarios are equal. The scenarios differed substantially, however, in the speed of take-up of clean cook stoves, which makes that a key component of the model, far more influential than clean lighting in determining future differences between scenarios. In terms of model validation, however, our simulation only manages to capture the general trend for this variable, and therefore the fit could not be described as an exceptionally good fit (Fig. 2, upper section). Replication of historical data is a key behavioural test for the validation of system dynamics models. Therefore, the model's inability to capture historical developments closely enough entails further caution in using this analysis as the only input to policymaking.

Policies for decreasing household air pollution are certainly not limited to those investigated in this paper. Behavioural change interventions, for instance, could succeed in moving households from cooking inside their rooms to cooking outdoors. Such behavioural policies are not considered within the scope of this study.

Another limitation of this study is the exogenous treatment of the level of *outdoor air pollution*, which was included as a scenario variable whose future value is set by the model user. For a more realistic treatment of the problem of household air pollution, it would be useful to model outdoor air pollution as an endogenous variable that is a composite of pollution that originates indoors and diffuses locally, as well as industrial, waste and transport pollution. Endogenising *outdoor air pollution* presents a potentially fruitful opportunity for further research in this line.

When we asked workshop participants to articulate their hopes for Nairobi slums, only one participant mentioned the reduction of household air pollution. Other issues such as land ownership, services and waste management were much higher on people's agendas. Even though the air pollution-related health burden is known to be very large, to our knowledge there are currently no programmes focusing on air pollution from a health point of view, neither at national nor at county level. This lack of attention (Zimmermann et al., 2017) presents an interesting conundrum and another potentially fruitful area for further research.

This project and modelling work was influenced by the limited attention that household air pollution has received so far. Because of this inattention, very little data on household air pollution has been collected to date. Increased investments in researching household air pollution would lead to more abundant scientific evidence, which could be used to produce more useful and reliable policy recommendations.

## Figures and Tables

**Table A-1:** Model coding explained.

Code	Meaning
Lower-case variable	Endogenous variable (formulated based on other variables within the model). The dynamic behaviour of such variables is given by software simulation.
Upper-case variable	Constant. Such constants are either fixed parameters (black), or policy/scenario variables set by the user (green)
Variable with first word in upper case, rest in lower case	Exogenous (data) variable. Past behaviour of such variables is given by historical data. Variable stays constant for future simulation, unless otherwise specified.
Red variable	Key indicator.
Green variable	Policy/scenario variable, decided upon by the user.
Blue variable, in angle brackets	‘Shadow’ variable, copied from another section of the model.
Blue arrows	Causal relationships, from cause to effect. Each (endogenous) variable is formulated based on variables connected to it via incoming arrows.
Grey arrows	Initial condition setting.

**Fig. A-1:**
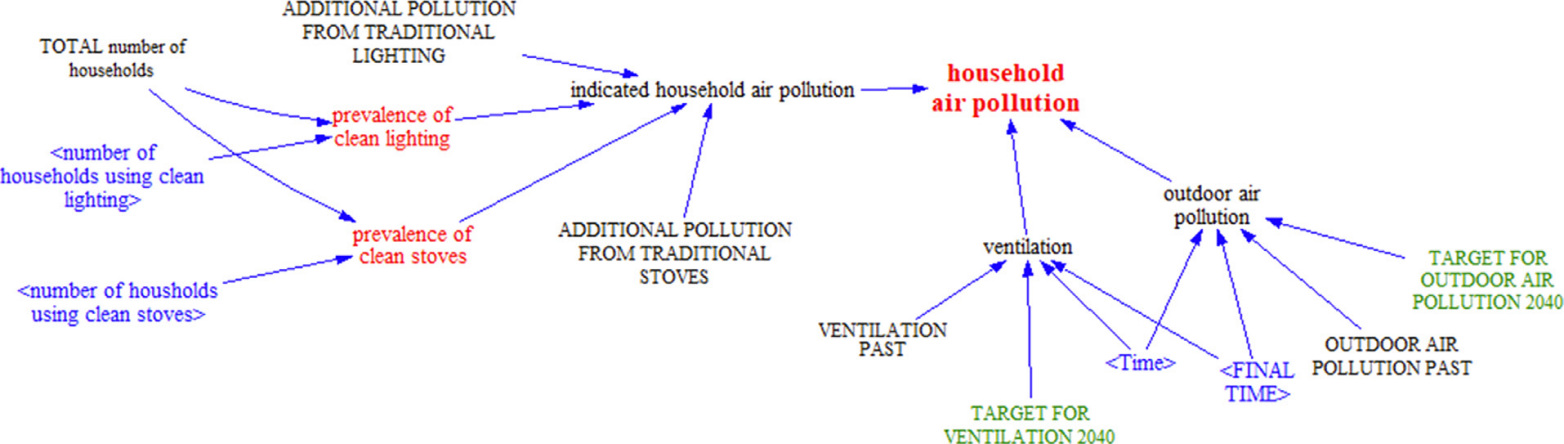
Formulation of household air pollution.

**Table A-2:** Distribution of households by cooking fuel type and mean PM_2.5_ levels.

Outcome	Korogocho (24)	Viwandani (48)	Total	Test statistic
Proportion of households using different cooking fuels (%)				χ^2^ (*p*-value) 24.6 (*p* < 0.001)
Charcoal or wood	[1] 62.5	[7] 14.6	[13] 30.6	
Kerosene	[2] 12.5	[8] 72.9	[14] 52.8	
LPG/electricity	[3] 25.0	[9] 12.5	[15] 16.7	
PM_2.5_ mean levels for different cooking fuels (μg/m^3^)				*t*-Statistic (*p*-value)
Charcoal or wood	[4] 126.5	[10] 75.7	[16] 110.0	6.59 (*p* < 0.001)
Kerosene	[5] 109.0	[11] 58.7	[17] 61.9	7.43(*p* < 0.001)
LPG/electricity	[6] 72.0	[12] 45.6	[18] 59.1	10.04 (*p* < 0.001)

**Fig. A-2:**
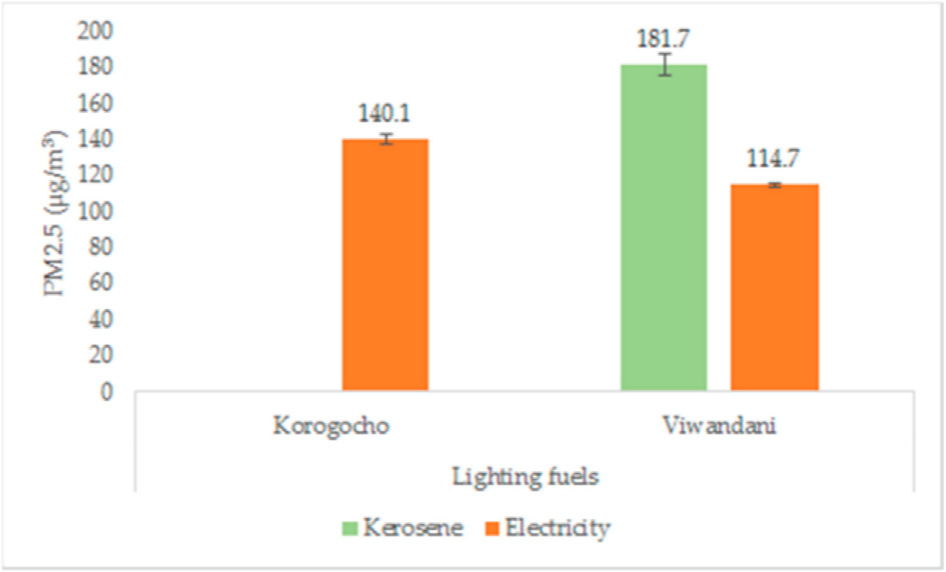
PM_2.5_ levels associated with lighting fuels.

Source: Muindi et al. (2016), Supplementary materials.

**Fig. A-3:**
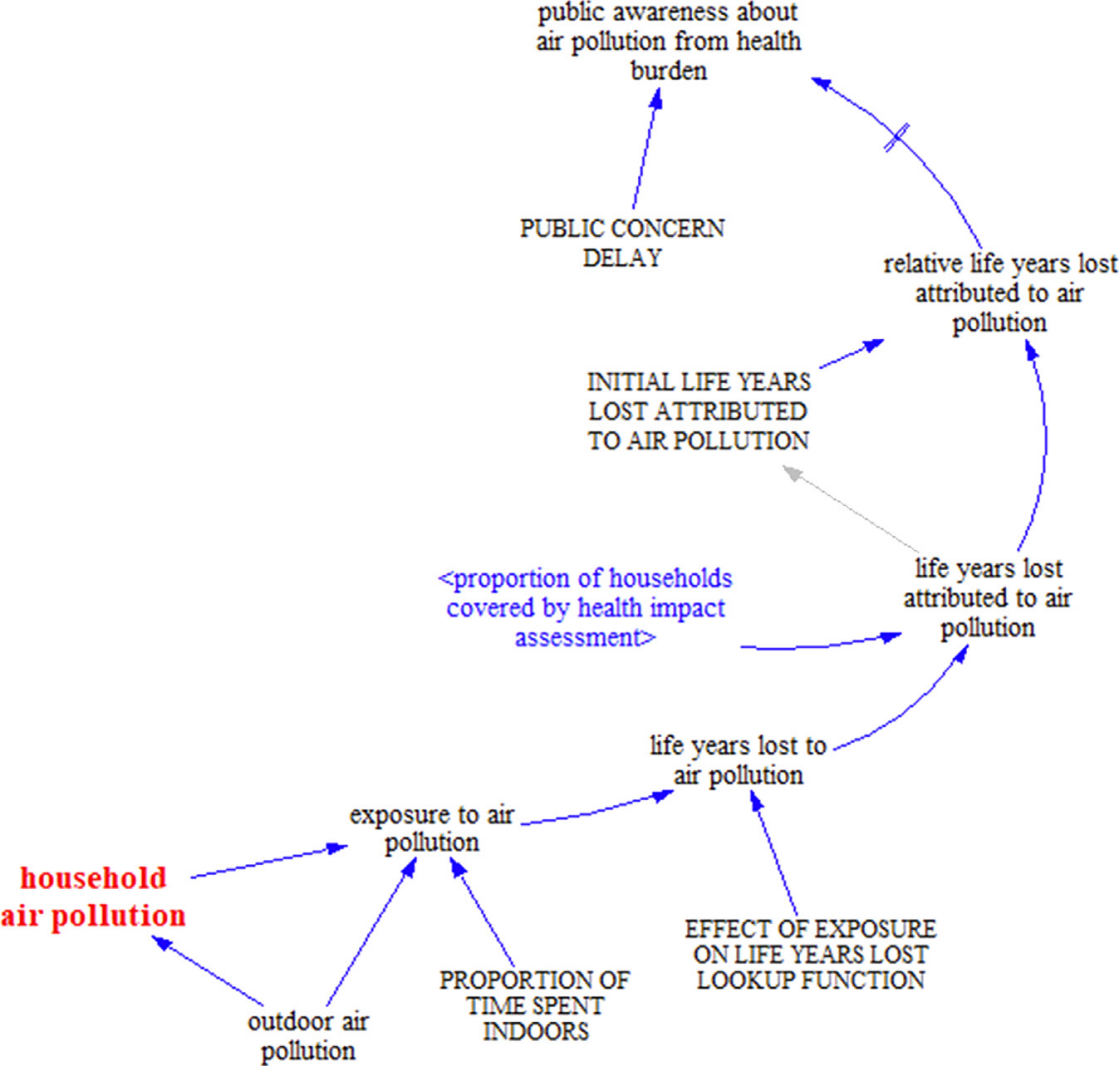
Formulation of life years lost to air pollution.

**Fig. A-4:**
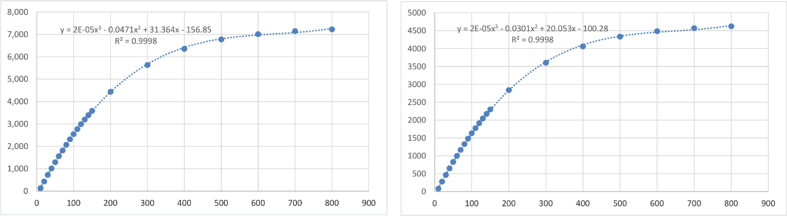
Health burden calculation. Exposure to air pollution on the x-axis and life years lost on the y-axis. Korogocho (left) and Viwandani (right).

**Fig. A-5:**
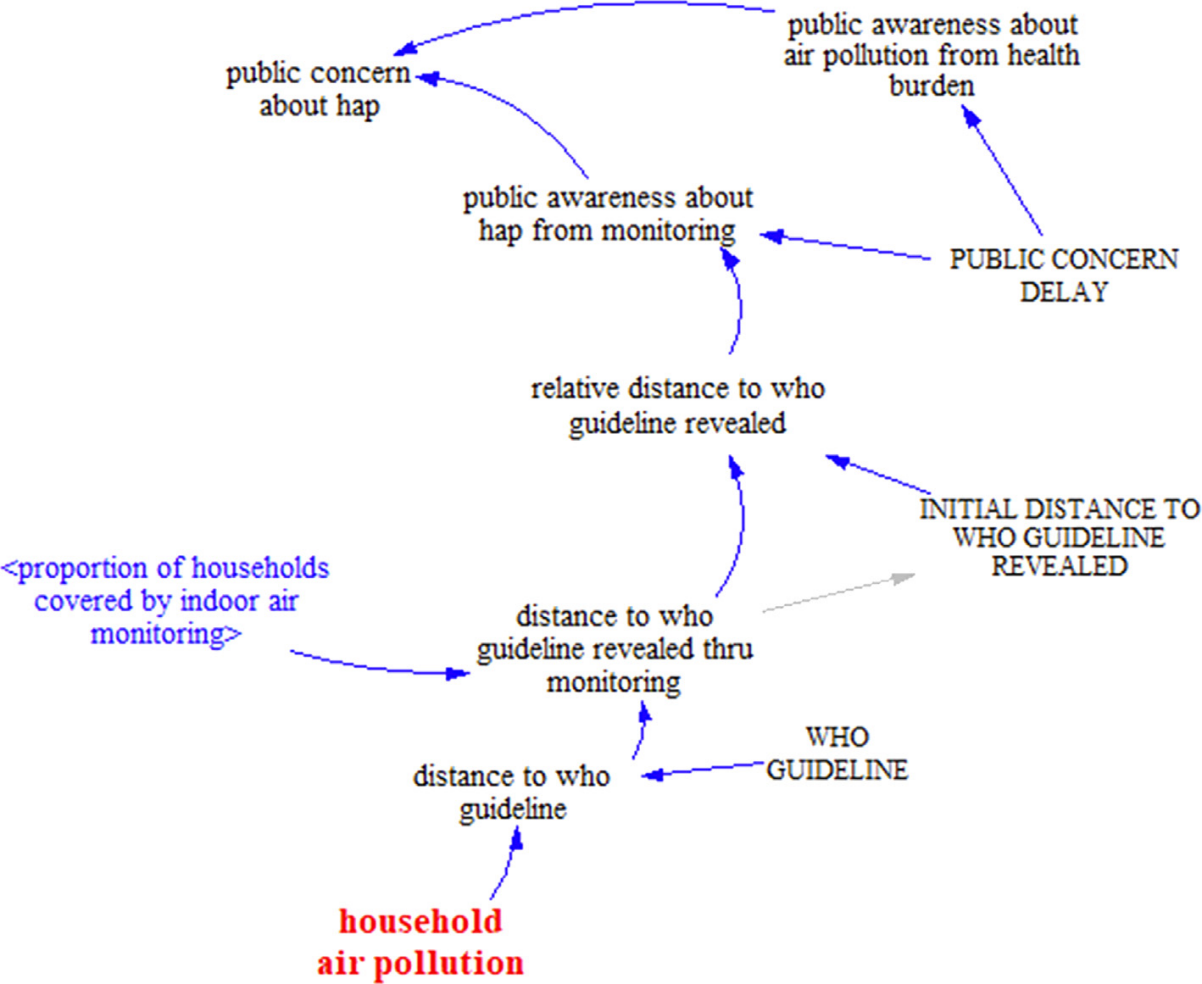
Formulation of Public concern about household air pollution.

**Fig. A-6:**
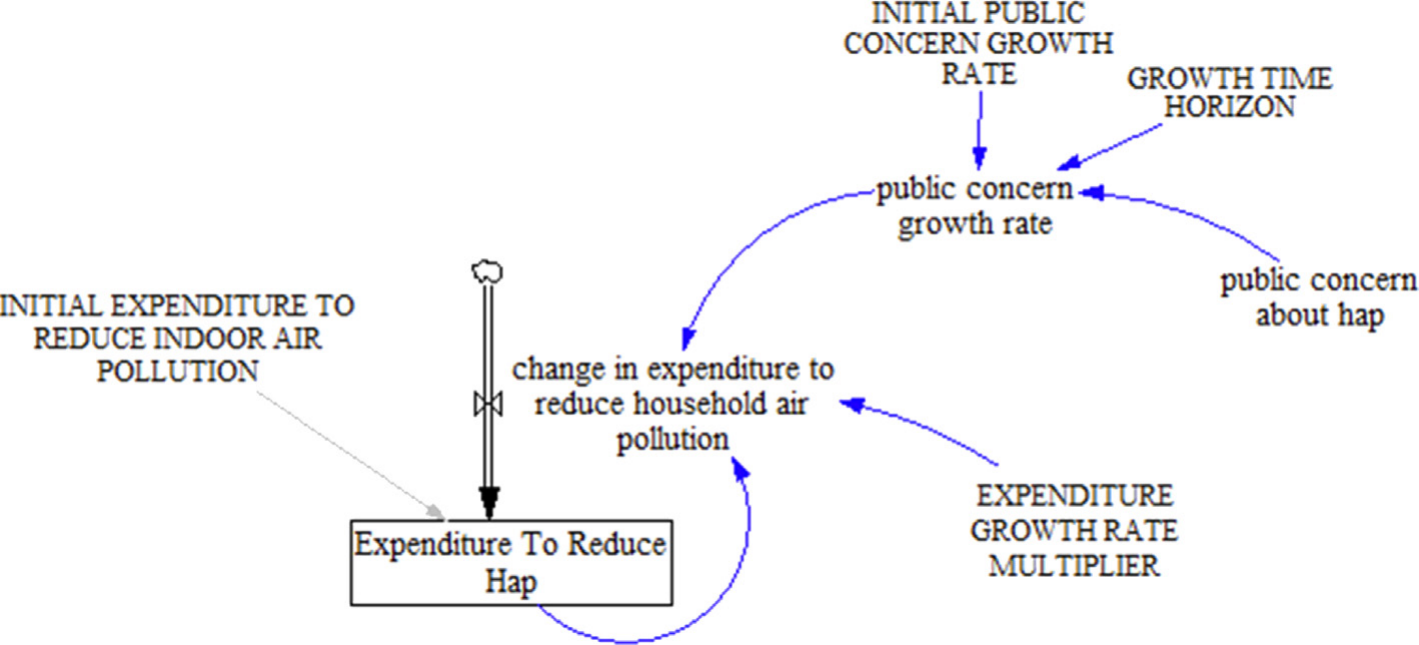
Formulation of expenditure.

**Fig. A-7:**
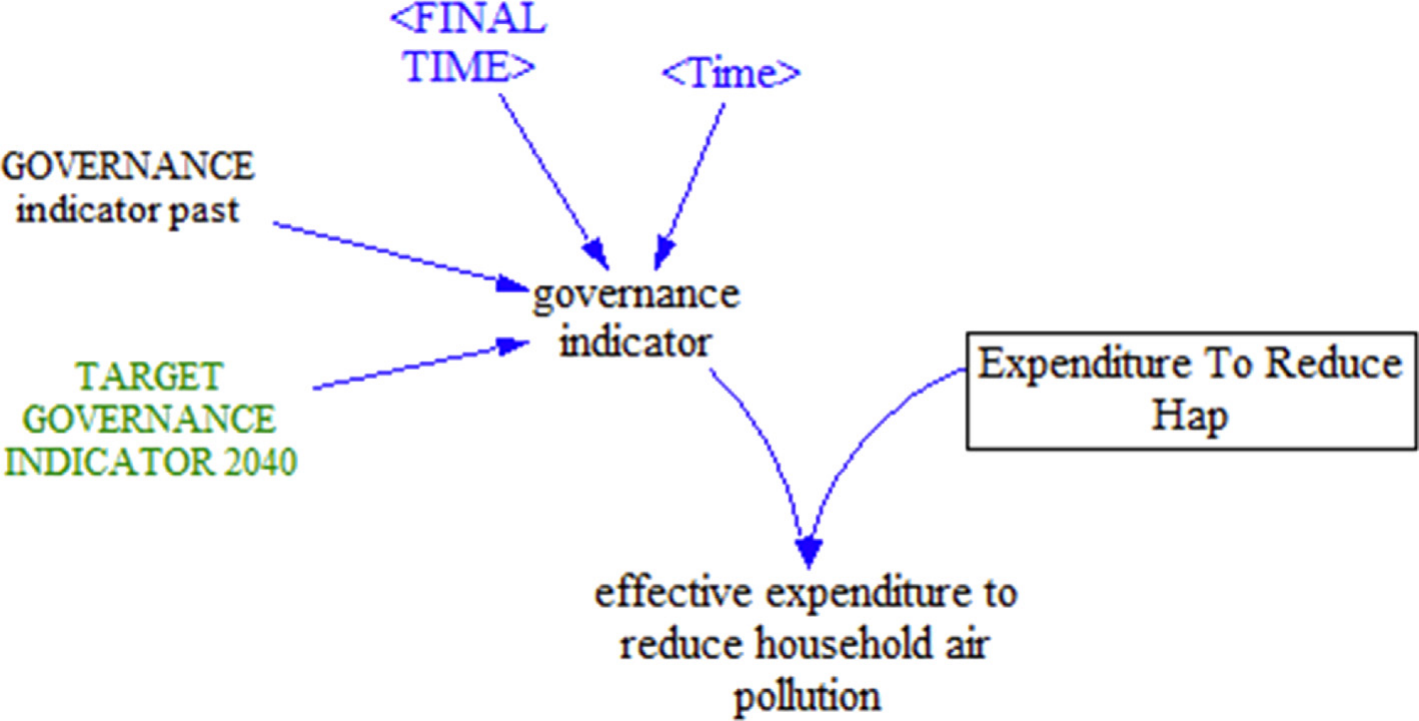
Formulation of good governance and effective expenditure.

**Fig. A-8:**
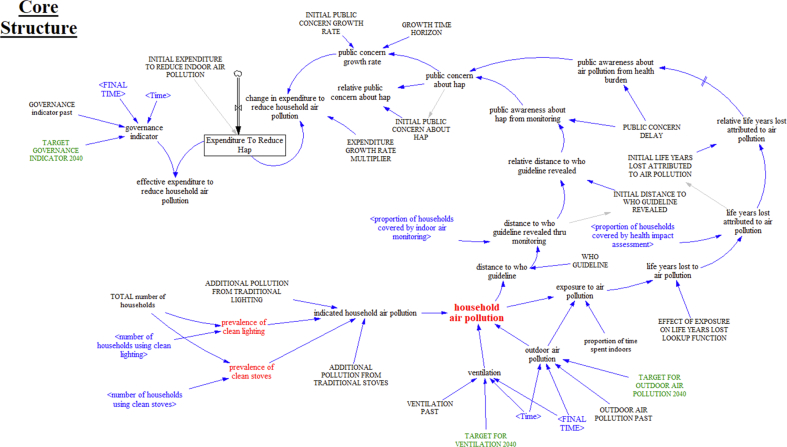
Core structure of the model.

**Fig. A-9:**
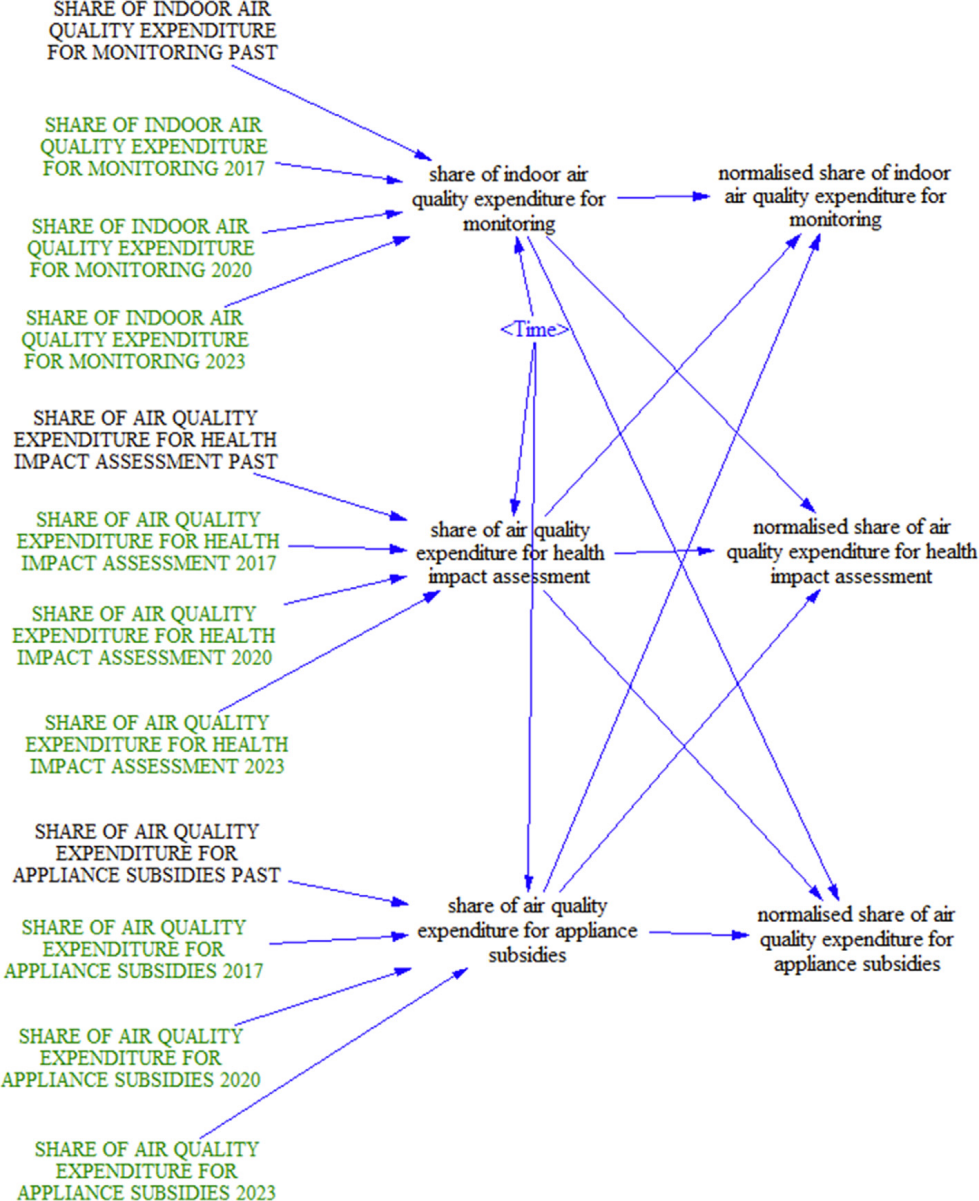
Division of funds.

**Fig. A-10:**
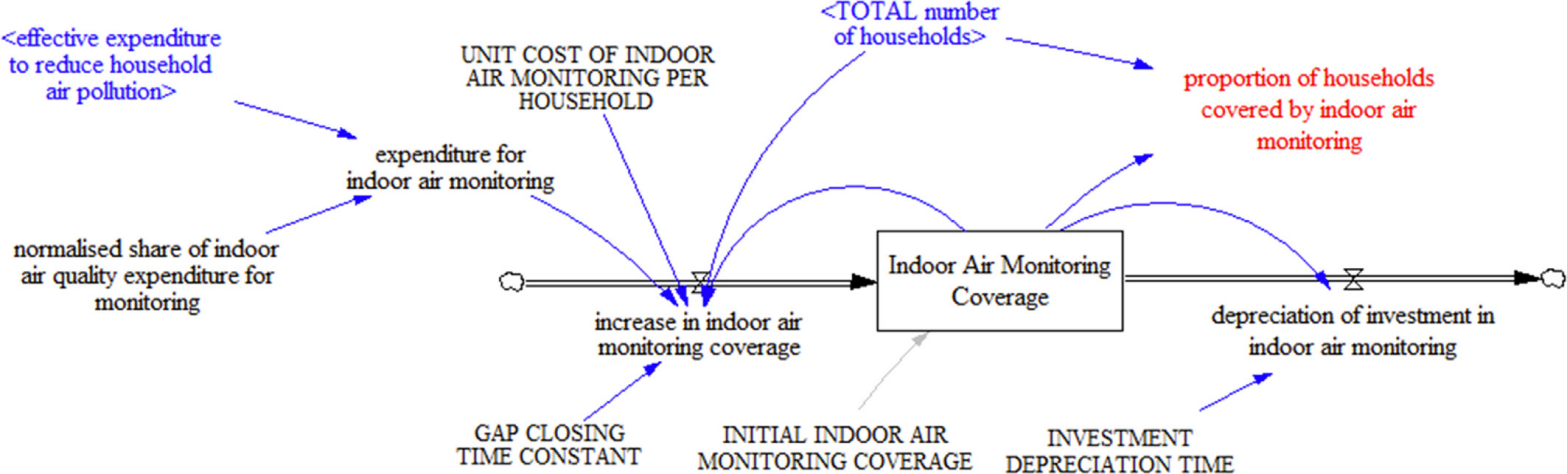
Formulation of indoor air monitoring coverage.

**Fig. A-11:**
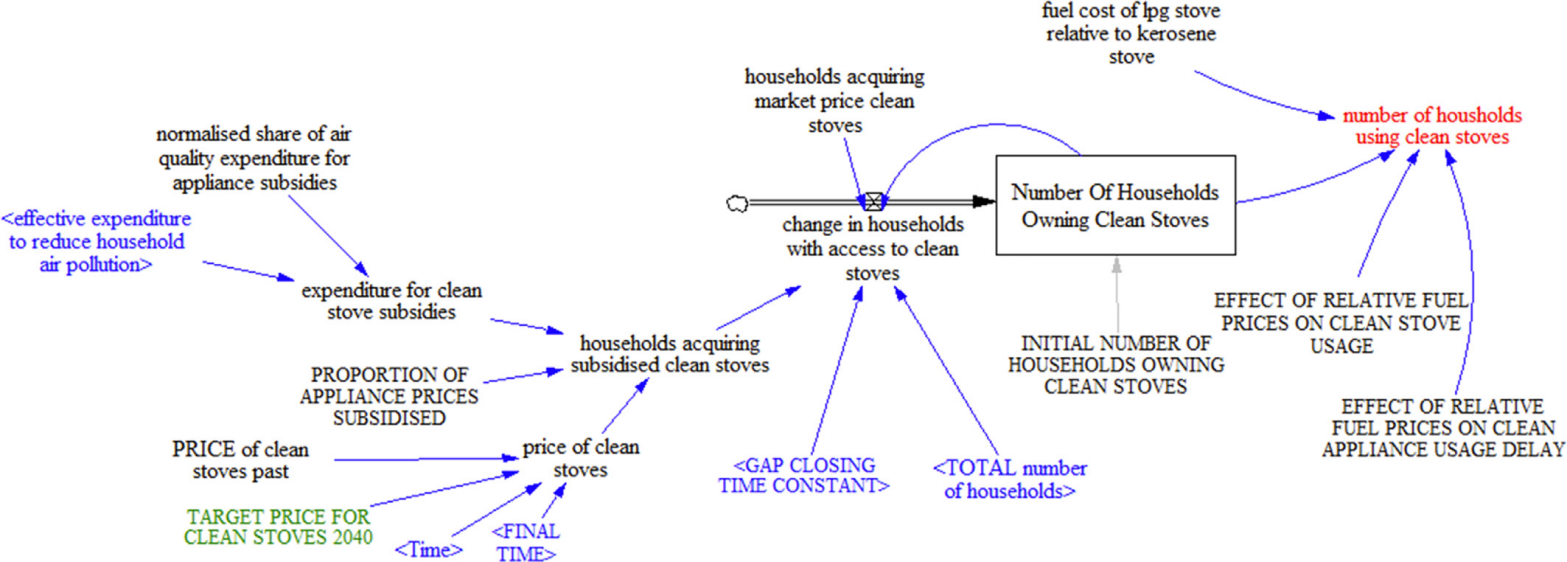
Formulation of number of households using clean stoves.

**Fig. A-12:**
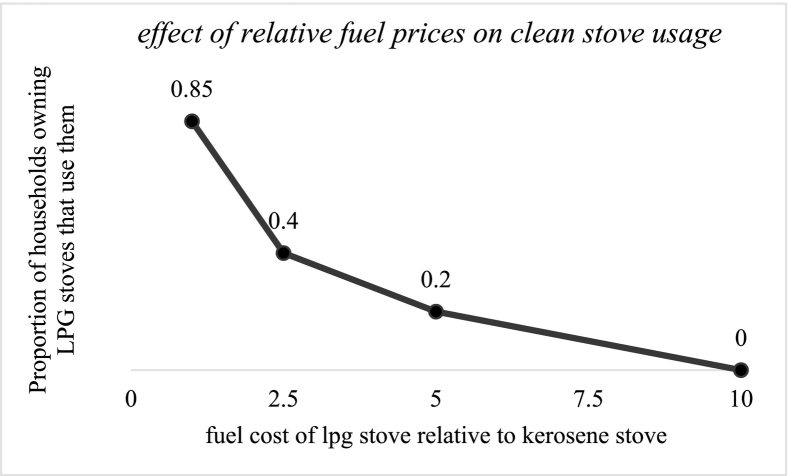
Effect of relative fuel prices on clean stove usage.

**Fig. A-13:**
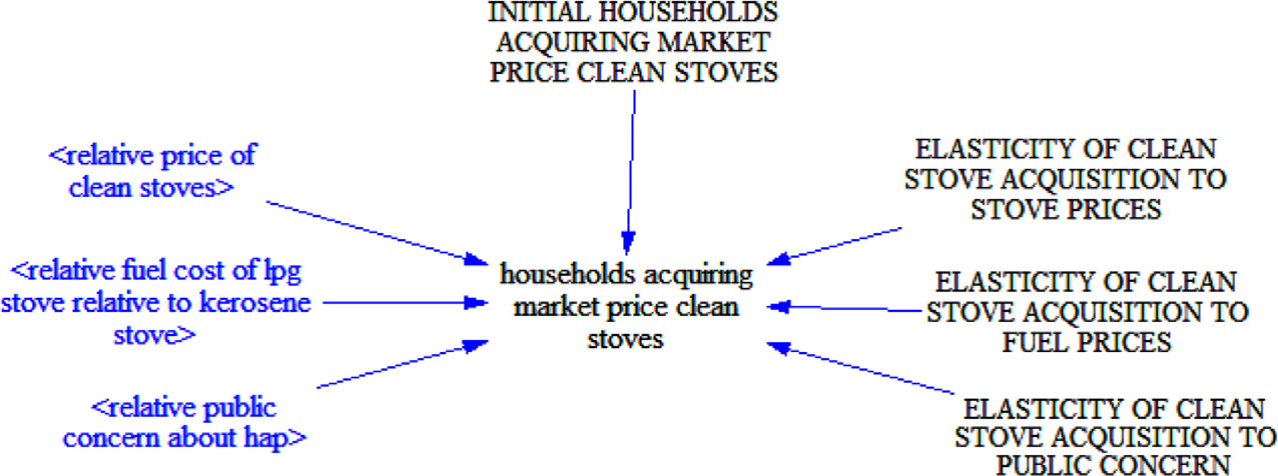
Formulation of households acquiring market price clean stoves.

**Fig. A-14:**
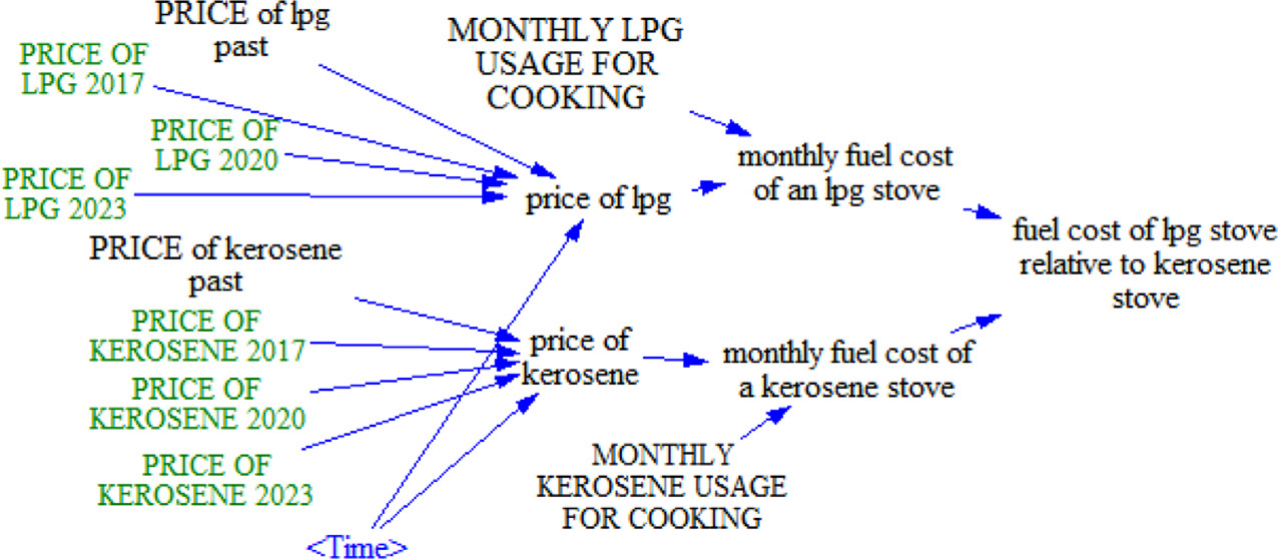
Formulation of fuel cost of LPG stove relative to kerosene stove.

**Fig. A-15:**
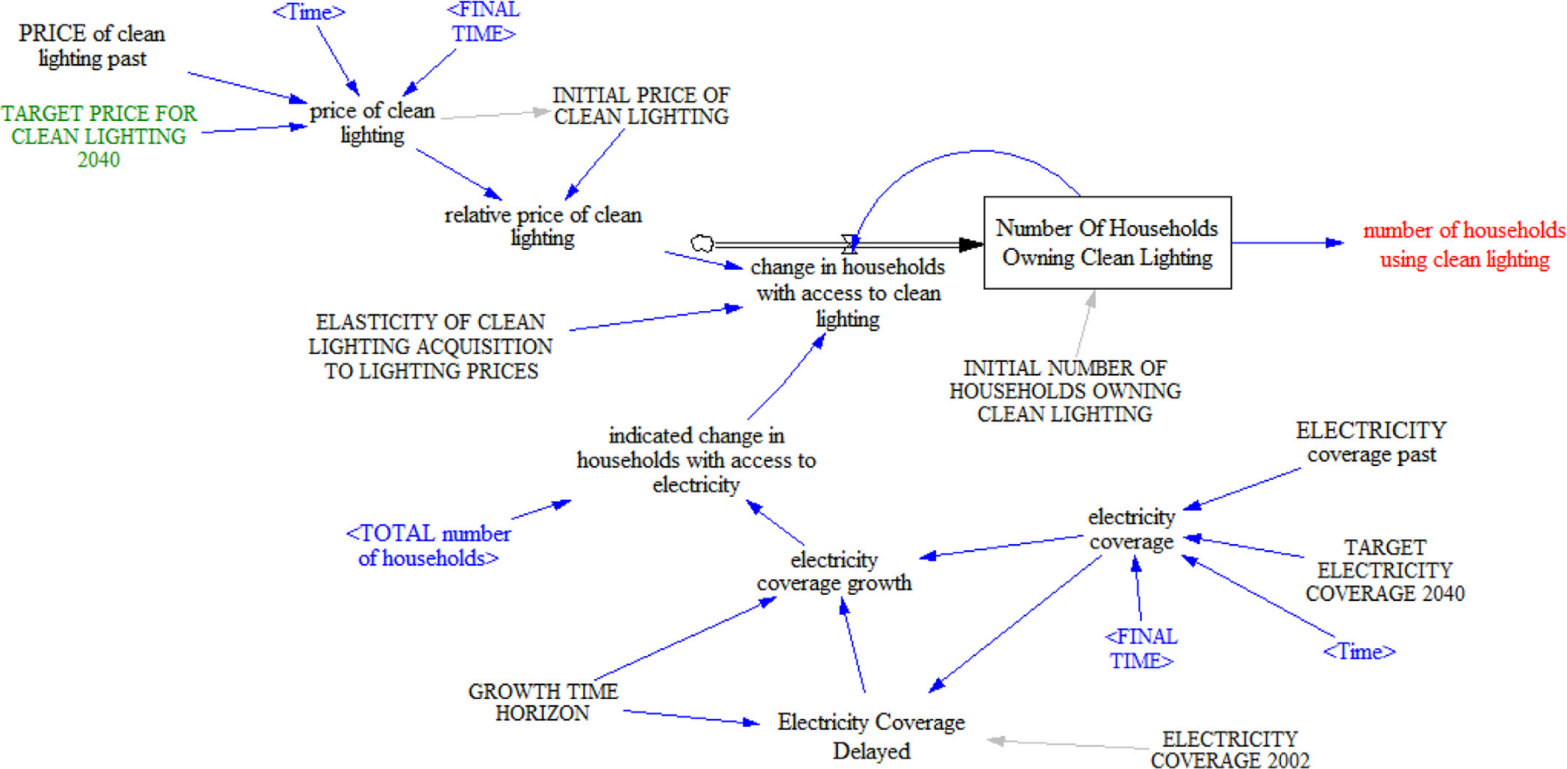
Formulation of number of households using clean lighting.

**Fig. A-16:**
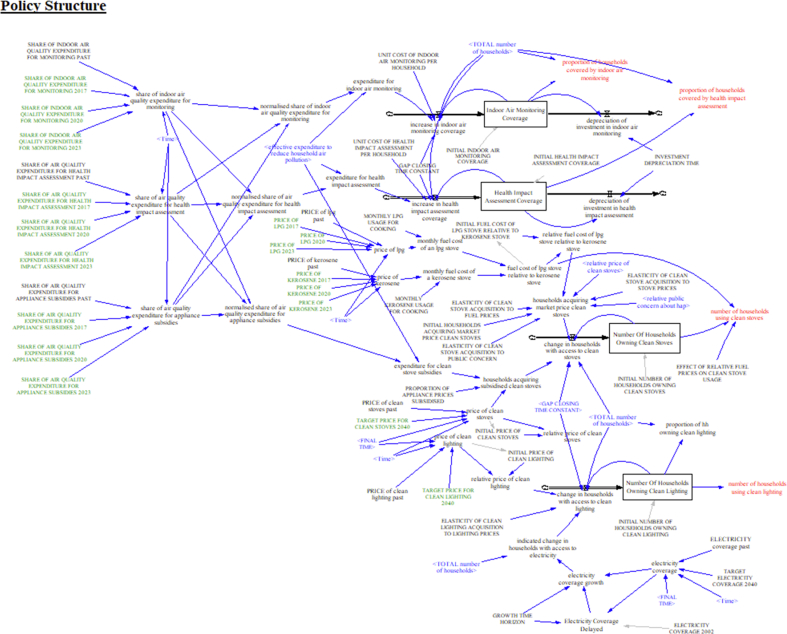
Policy structure.

**Fig. B-1:**
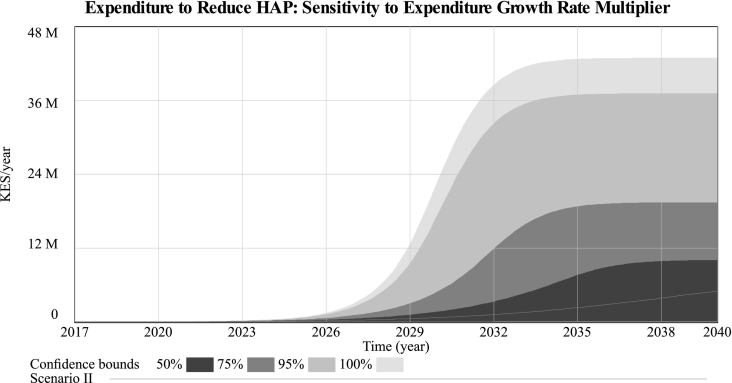
Sensitivity of expenditure to reduce household air pollution to expenditure growth rate multiplier.

**Fig. B-2:**
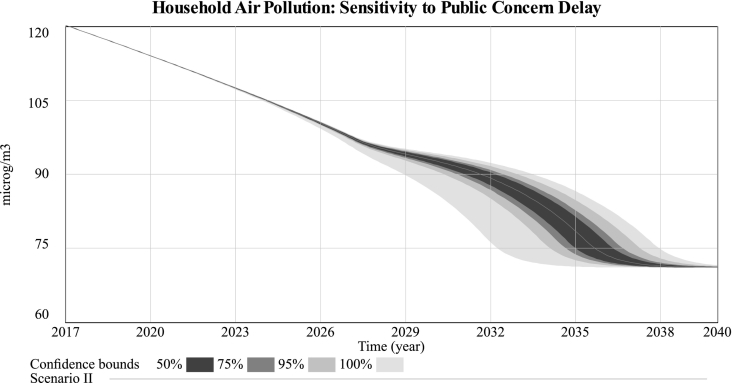
Sensitivity of household air pollution to public concern delay.

**Fig. B-3:**
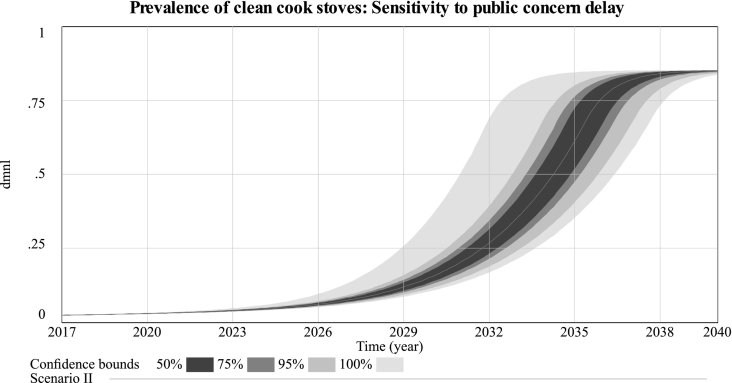
Sensitivity of prevalence of clean cook stoves to public concern delay.

**Fig. B-4:**
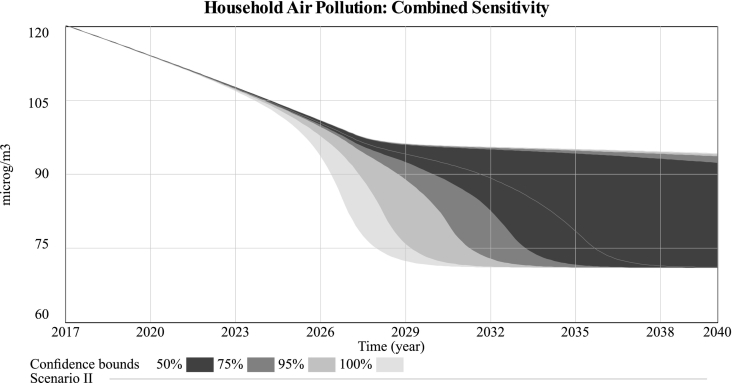
Sensitivity of household air pollution to public concern delay and expenditure growth rate multiplier.

**Table:** 

Policy/scenario variable	Unit	Base run	Scenario I	Scenario II	Scenario III
Future share of air quality expenditure for appliance subsidies	Dimensionless	98%	=	@2017: 90% @2020: 80% @2023: 70%	@2017: 90% @2020: 80% @2023: 70%
Future share of indoor air quality expenditure for monitoring	Dimensionless	1%	=	@2017: 5% @2020: 10% @2023: 15%	@2017: 5% @2020: 10% @2023: 15%
Future share of air quality expenditure for health impact assessment	Dimensionless	1%	=	@2017: 5% @2020: 10% @2023: 15%	@2017: 5% @2020: 10% @2023: 15%
Future price of kerosene	KSH per litre	63.4	@2017: 70 @2020: 80 @2023: 90	@2017: 70 @2020: 80 @2023: 90	@2017: 70 @2020: 80 @2023: 90
Future price of LPG	KSH per 6 kg cylinder	1246	@2017: 1160 @2020: 1080 @2023: 1000	@2017: 1160 @2020: 1080 @2023: 1000	@2017: 1160 @2020: 1080 @2023: 1000
Target for price of clean stoves 2040	KSH per unit	2000	By 2040 linearly down to 1000	By 2040 linearly down to 1000	By 2040 linearly down to 1000
Target for price of clean lighting 2040	KSH per unit	1000	By 2040 linearly down to 500	By 2040 linearly down to 500	By 2040 linearly down to 500
Target for good governance 2040	Dimensionless (conceptualised as a 0 to 1 index)	0.383	By 2040 linearly up 50% to 0.575	By 2040 linearly up 50% to 0.575	By 2040 linearly up 50% to 0.575
Target for ventilation 2040 (only Korogocho)	Dimensionless	0.4	=	=	By 2040 linearly up to 0.6
Target for outdoor air pollution 2040	Microgram/cubic metre	166	=	=	By 2040 linearly down to 83 (Korogocho) and 33.5 (Viwandani)

**Table:** 

Parameter name	Value (Korogocho model)	Value (Viwandani model)	Source	Note
Additional pollution from traditional lighting	67 [μg/m^3^]	=	Estimated based on Muindi et al. (2016)	See Appendix A.1.1
Additional pollution from traditional stoves	51.7 [μg/m^3^]	15.9 [μg/m^3^]	Estimated based on Muindi et al. (2016)	See Appendix A.1.1
Elasticity of clean lighting acquisition to lighting prices	−0.5	=	Obtained through calibration	Calibrated to historical data on prevalence of clean lighting
Elasticity of clean stove acquisition to fuel prices	−0.3	=	Obtained through calibration	Calibrated to historical data on prevalence of clean stoves
Elasticity of clean stove acquisition to public concern	0.2	=	Obtained through calibration	Calibrated to historical data on prevalence of clean stoves
Elasticity of clean stove acquisition to stove prices	−0.4	=	Obtained through calibration	Calibrated to historical data on prevalence of clean stoves
Electricity coverage 2002	0.01	=	Estimated by extrapolating into the past the electricity coverage in 2003 and 2004	NUHDSS data on households supplied with electricity from the national grid
Expenditure growth rate multiplier	0.75	=	Assumption	See Appendix B.1 for an analysis of sensitivity to this assumption
Gap closing time constant	1 [year]	=	Assumption	Standard system dynamics modelling practice
Growth time horizon	0.5 [year]	=	Assumption	Measuring half-yearly growth rate in electricity coverage
Initial expenditure to reduce indoor air pollution	9500 [KSH/year]	12,500 [KSH/year]	Estimation	See Appendix A.1.4
Initial health impact assessment coverage	1	=	Assessment of APHRC co-authors	Virtually non-existent HIA in 2003. See Appendix A.2.2
Initial indoor air monitoring coverage	1	=	Assessment of APHRC co-authors	Virtually non-existent monitoring in 2003. See Appendix A.2.2
Initial households acquiring market price clean stoves	12 [households/year]	21 [households/year]	Obtained through calibration	Calibrated to historical data on prevalence of clean stoves
Initial number of households owning clean lighting	148 [households]	221 [households]	NUHDSS	Nairobi Urban Health and Demographic Surveillance System
Initial number of households owning clean stoves	6 [households]	12 [households]	NUHDSS	
Initial public concern growth rate	0	=	Assumption	Representing the initial non-existence of HIA or monitoring initiatives
Investment depreciation time	5 [years]	=	Assumption	
Monthly kerosene usage for cooking	24 [litre/month]	=	Price data from the Kenyan National Bureau of Statistics (KNBS). Usage data from residents' estimates	See Appendix A.2.3
Monthly LPG usage for cooking	0.33 [canister/month]	=	Price data, see below. Usage data from residents' estimates.	
Outdoor air pollution past	166 [μg/m^3^]	67 [μg/m^3^]	Egondi, Muindi, Kyobutungi, Gatari, & Rocklöv (2016)	
Price of kerosene past	63.4 [KSH/litre]	=	Prices regulated by the Energy Regulatory Authority	http://www.erc.go.ke/index.php?option=com_content&view=article&id=162&Itemid=666
Price of LPG past	1246 [KSH/canister]	=	Kenya National Bureau of Statistics (KNBS) and local newspapers	https://www.knbs.or.ke/data-releases/
Proportion of appliance prices subsidised	0.4	=	Assumption	This assumption was validated against expert judgment of APHRC co-authors.
Public concern delay	2 [years]	=	Assumption	See Appendix B.2 for an analysis of sensitivity to this assumption
Share of air quality expenditure for appliance subsidies past	0.98	=	Assumption	Representing the allocation of virtually all resources to subsidising appliances in the past
Share of air quality expenditure for health impact assessment past	0.01	=	Assumption	Representing the allocation of virtually no resources to HIA in the past
Share of indoor air quality expenditure for monitoring past	0.01	=	Assumption	Representing the allocation of virtually no resources to monitoring in the past
Target electricity coverage 2040	1	=	See footnote 2	Full coverage assumed by 2040
Unit cost of health impact assessment per household	100 [KSH/household]	=	Appraisal made by co-authors from APHRC	
Unit cost of indoor air monitoring per household	100 [KSH/household]	=	Appraisal made by co-authors from APHRC	
Ventilation past	0.4	0.68	Estimation based on Muindi et al. (2016)	See Appendix A.1.1
WHO guideline	10 [μg/m^3^]	=	(World Health Organization, 2006)	

## Appendix A Model documentation

In this appendix, the formulation and parametrisation of the system dynamics model is explained in detail. The model is built in the well-established system dynamics simulation software *Vensim*. The whole 150-plus-variable model is presented in smaller pieces, with visual snapshots to aid understanding. The following table elaborates the colour-coding and other information needed to interpret the diagrams.

We have parametrised two versions of the model, one for each one of the two slums under study, Korogocho and Viwandani. The two models are identical in structure but parametrised differently to reflect the conditions in each setting. Therefore, each of the two models is an aggregate representation of one of the two slums, and when, for instance, we talk about *household air pollution*, we refer to the ‘average’ household within the context.

The model is comprised of two sectors (or ‘views’ as they are called in the Vensim software): The *Core Structure*, and the *Policy Structure*. Below we start with a detailed explanation of formulation of the *Core Structure*, which is followed with the *Policy Structure* being elaborated next.

In documenting equations, units are indicated in brackets. Where no units are indicated, the variable is dimensionless.

### A.1 Core structure

#### A.1.1 Household air pollution

Fig. A-1 portrays the formulation of our central indicator, *household air pollution* (sometimes abbreviated to *HAP*). *Household air pollution* is the weighted average of *indicated household air pollution* and *outdoor air pollution*. The weight is given by the extent of *ventilation*.

$$\begin{array}{l} \mathrm{household}\;\mathrm{air}\;\mathrm{pollution}\;\left[\mathrm{\mu g}/m^{3}]=\mathrm{indicated household}\;\mathrm{air}\;\mathrm{pollution}\;[\mathrm{\mu g}/m^{3}\right]*(1-\\ \mathrm{ventilation})+\mathrm{outdoor}\;\mathrm{air}\;\mathrm{pollution}\;\left[\mathrm{\mu g}/m^{3}\right]*\mathrm{ventilation} \end{array}$$

*Outdoor air pollution*, *proxied by the concentration of PM*_2.5_, is assumed to be constant in the past,^4^ set to 166 μg/m^3^ for Korogocho and 67 μg/m^3^ for Viwandani, based on a single data point available from Egondi et al. (2016). For the future, a *target for outdoor air pollution* can be set by the user for the year 2040 (*Final Time* of simulation). The following formulation will then linearly change current *outdoor air pollution* towards the set target^5^:

$$\begin{array}{l} \mathrm{Outdoor}\;\mathrm{air}\;\mathrm{pollution}\;\left[\mathrm{\mu g}/m^{3}\right]=\mathrm{IF THEN ELSE}\;(\mathrm{Time}<2017,\mathrm{OUTDOOR}\;\mathrm{AIR}\\ \mathrm{POLLUTION PAST}\;\left[\mathrm{\mu g}/m^{3}\right],\\ \mathrm{OUTDOOR}\;\mathrm{AIR}\;\mathrm{POLLUTION PAST}\;\left[\mathrm{\mu g}/m^{3}\right]+(\mathrm{TARGET FOR OUTDOOR}\;\mathrm{AIR}\\ \mathrm{POLLUTION}\;2040\;\left[\mathrm{\mu g}/m^{3}\right]-\mathrm{OUTDOOR}\;\mathrm{AIR}\;\mathrm{POLLUTION PAST}\;\left[\mathrm{\mu g}/m^{3}\right])*(\mathrm{Time}-\\ 2017)/\left(\mathrm{FINAL TIME}-2017\right)) \end{array}$$

*Ventilation* is similarly assumed to be constant in the past, with a future target set by the user. *Ventilation* is defined as an index between zero and 1, where a value of zero (no ventilation) would denote that outdoor air has no effect on *household air pollution*, and 1 (full ventilation – no walls around) would mean that *household air pollution* is entirely determined by *outdoor air pollution*. The past value is obtained deriving information from Muindi et al. (2016). In Muindi et al.'s paper (Table A-2), PM_2.5_ levels for households using LPG/electricity is given as 72.0 [μg/m^3^] for Korogocho and 45.6 [μg/m^3^] for Viwandani. Since these household are using clean fuels, we assume that this pollution is coming from outdoors. Therefore, we can obtain a very rough estimate for *ventilation past* by dividing the given levels of *household air pollution* for households using clean fuels by the level of *outdoor air pollution*. (*Ventilation past* for Korogocho = 72/166 = 0.4. *Ventilation past* for Viwandani = 45.6/67 = 0.68).

*Indicated household air pollution* in our model comes from two sources: cooking and lighting. Pollution from these two sources is added together.

The contribution of each source is calculated as the typical pollution resulting from using traditional appliances (*additional pollution from traditional stoves*/lighting) times the prevalence of those appliances, i.e. *1 minus the prevalence of clean stoves*/*lighting*.

$$\begin{array}{l} \mathrm{Indicated household}\;\mathrm{air}\;\mathrm{pollution}\;\left[\mathrm{\mu g}/m^{3}\right]=\\ \left(1-\mathrm{prevalence of clean stoves}\right)*\mathrm{ADDITIONAL POLLUTION FROM TRADITIONAL}\\ \mathrm{STOVES}\;\left[\mathrm{\mu g}/m^{3}\right]+\\ \left(1-\mathrm{prevalence of clean lighting}\right)*\mathrm{ADDITIONAL POLLUTION FROM TRADITIONAL}\\ \mathrm{LIGHTING}\;\left[\mathrm{\mu g}/m^{3}\right] \end{array}$$

*Additional pollution from traditional stoves*/*lighting* are two parameters which are estimated based on Muindi et al. (2016) by subtracting the level of *household air pollution* in a household using clean fuels from that of a household using dirty fuels, assuming the difference to be attributable to the use of traditional stoves.

The level of *household air pollution* in households using dirty fuels is calculated as the weighted average of those using kerosene and those using charcoal/wood. For Korogocho, according to the numbers in Table A-2, this is:

$$\begin{array}{l} \left[4\right]*\left[1\right]/\left(\left[1\right]+\left[2\right]\right)+\left[5\right]*\left[2\right]/\left(\left[1\right]+\left[2\right]\right)=\\ 126.5\;\left[\mathrm{\mu g}/m^{3}\right]*62.5\%/\left(62.5\%+12.5\%\right)+109.6\;\left[\mathrm{\mu g}/m^{3}\right]*12.5\%/\left(62.5\%+12.5\%\right)=123.7\\ \left[\mathrm{\mu g}/m^{3}\right]. \end{array}$$

And for Viwandani:

$$\begin{array}{l} \left[10\right]*\left[7\right]/\left(\left[7\right]+\left[8\right]\right)+\left[11\right]*\left[8\right]/\left(\left[7\right]+\left[8\right]\right)=\\ 75.7\;\left[\mathrm{\mu g}/m^{3}\right]*14.6\%/\left(14.6\%+72.9\%\right)+58.7\;\left[\mathrm{\mu g}/m^{3}\right]*72.9\%/\left(14.6\%+72.9\%\right)=61.5\\ \left[\mathrm{\mu g}/m^{3}\right]. \end{array}$$

Therefore, the difference between households using dirty fuels with those using clean fuels (LPG/electricity, [6] and [12] in the table above) is given for each setting:

$$\begin{array}{l} \left[\text{Korogocho}\right]\;\mathrm{additional pollution from traditional stoves}=123.7\;\left[\mathrm{\mu g}/m^{3}\right]-72\;\left[\mathrm{\mu g}/m^{3}\right]\\ =51.7\;\left[\mathrm{\mu g}/m^{3}\right].\\ \left[\text{Viwandani}\right]\;\mathrm{additional pollution from traditional stoves}=61.5\;\left[\mathrm{\mu g}/m^{3}\right]-45.6\\ \left[\mathrm{\mu g}/m^{3}\right]=15.9\;\mathrm{\mu g}/m^{3}. \end{array}$$

We attribute these figures to the use of non-clean stoves, and we use the above values for *additional pollution from traditional stoves* in each setting. This method of estimation gives rather different values for additional pollution from traditional stoves for the two slums. This may be because in Korogocho the population predominantly uses charcoal, while in Viwandani households mostly use kerosene. A useful extension of this study could be towards disaggregating the representation of these fuels in the model.

As for lighting, we use a similar method based on the following diagram from the same article (Muindi et al., 2016). In this case, data for Korogocho is incomplete (Fig. A-2), so we use the parameter obtained for Viwandani in both models:

$$\begin{array}{l} \mathrm{additional pollution from traditional lighting}=181.7\;\left[\mathrm{\mu g}/m^{3}\right]–114.7\;\left[\mathrm{\mu g}/m^{3}\right]=67\;\\ \left[\mathrm{\mu g}/m^{3}\right]. \end{array}$$

*Prevalence of clean lighting*/*stoves* is defined as the *number of households using clean lighting*/*stoves* divided by *total number of households*. The latter is a data series available from the Nairobi Urban Health Demographic Surveillance System (NUHDSS) (Emina et al., 2011). The *prevalence* variables are shadow variables that are copied from another section of the model, which we will describe later in this appendix (see Appendix A.2.3).

#### A.1.2 Health burden and public awareness

Next, we describe how we capture *life year lost to air pollution* and *public awareness* about it in our model (Fig. A-3).

*Exposure to air pollution* is calculated as a weighted average of *household air pollution* and *outdoor air pollution*, using *proportion of time spent indoors* as the weighting factor.

$$\begin{array}{l} \mathrm{Exposure to}\;\mathrm{air}\;\mathrm{pollution}\;\left[\mathrm{\mu g}/m^{3}\right]=\\ \mathrm{household}\;\mathrm{air}\;\mathrm{pollution}\;\left[\mathrm{\mu g}/m^{3}\right]*\mathrm{PROPORTION OF TIME SPENT INDOORS}\\ +\mathrm{outdoor}\;\mathrm{air}\;\mathrm{pollution}\;\left[\mathrm{\mu g}/m^{3}\right]*\left(1-\mathrm{PROPORTION OF TIME SPENT INDOORS}\right) \end{array}$$

It is assumed that the population of the slum, on average, spends two thirds of their time indoors. This is a rough estimate given by local experts from APHRC. Obviously, this is a simplification, as this parameter would be different for different subsets of the population, e.g. children, women, etc. However, this simplification corresponds to the high-level aggregate view we take in our model. Changes in this parameter would affect *life years lost to air pollution*, but it would not change the general patterns of behaviour of other key indicators.

*Life years lost to air pollution* is obtained from *exposure to air pollution* using a life table model, as explained in the Methods section of this article (see Section 2.3) (Fig. A-4).

Thereafter, *life years lost attributed to air pollution* is proxied by the former variable multiplied by *proportion of households covered by health impact assessment*, which is a shadow variable copied from another section of the model which we will describe later (see Appendix A.2.2). In other words, it is assumed that the health burden of air pollution is correctly attributed to its cause only to the extent that the population is covered by health impact assessment studies.

$$\begin{array}{l} \mathrm{life years lost attributed to}\;\mathrm{air}\;\mathrm{pollution}\;\left[\mathrm{year}\right]=\\ \mathrm{life years lost to}\;\mathrm{air}\;\mathrm{pollution}\;\left[\mathrm{year}\right]*\mathrm{proportion of households covered}\;\mathrm{by}\;\mathrm{health}\;\mathrm{im}‐\\ \mathrm{pact assessment} \end{array}$$

Afterwards, this latter variable is normalized by dividing it by its *initial* value and named *relative life years lost attributed to air pollution*. Normalization allows us to arrive at a dimensionless indicator of the perceived air pollution related health burden in a respective settlement, which takes a value of unity at the beginning of the simulation, and which allows summation with *public awareness about air pollution from monitoring*, explained in the next section. An exponential smooth (i.e. delayed version) of this variable is taken as a proxy for *public awareness about air pollution from health burden* (a dimensionless variable). The delay is formulated as a third-order exponential smooth with a time constant of two years (*public concern delay*), using the software's built-in *SMOOTH3* function.^6^ This assumption implies that it takes on average about two years for results of health impact assessment studies to translate into public awareness. Sensitivity analysis on this uncertain parameter is carried out and reported on in Appendix B.2.

$$\begin{array}{l} \mathrm{public awareness about}\;\mathrm{air}\;\mathrm{pollution from health burden}=\\ \mathrm{SMOOTH}3(\mathrm{relative life years lost attributed to}\;\mathrm{air}\;\mathrm{pollution},\mathrm{PUBLIC CONCERN}\;\mathrm{DE}‐\\ \mathrm{LAY}) \end{array}$$

#### A.1.3 Monitoring and public awareness

Health impact assessment is not the only way to create awareness about household air pollution within the population. Awareness could also be raised by directly monitoring household air pollution (Fig. A-5).

To model the role of monitoring, first we measure the *distance* [of *household air* pollution] to *WHO guideline* of 10 μg/m^3^, reflecting the assumption that public concern in this case would be a function of this gap.

distance towhoguideline=householdairpollution−WHOGUIDELINE

Subsequently, we assume that this gap is only revealed to the extent that indoor air monitoring is carried out within the population. Therefore, in line with our method used above for health impact assessment:

$$\begin{array}{l} \mathrm{distance to}\;\mathrm{who}\;\mathrm{guideline revealed through monitoring}=\\ \mathrm{distance to}\;\mathrm{who}\;\mathrm{guideline}*\mathrm{proportion of households covered}\;\mathrm{by}\;\mathrm{indoor}\;\mathrm{air}\;\mathrm{monitoring} \end{array}$$

*Proportion of households covered by health impact assessment* is formulated in Appendix A.2.2. This latter variable is then normalized as above to arrive at *relative distance to WHO guideline revealed*. Thereafter, *public awareness about HAP from monitoring* is an exponential smooth of this latter variable, obtained in the same way as for health impact assessment.

$$\begin{array}{l} \mathrm{public awareness about}\;\mathrm{HAP}\;\mathrm{from monitoring}=\\ \mathrm{SMOOTH}3\left(\mathrm{relative distance to}\;\mathrm{who}\;\mathrm{guideline revealed},\mathrm{PUBLIC CONCERN DELAY}\right) \end{array}$$

Finally, *public concern about HAP* is simply the sum of the *awareness* coming from the two sources, previously made comparable through normalisation.

$$\begin{array}{l} \mathrm{public concern about}\;\mathrm{HAP}=\mathrm{public awareness about}\;\mathrm{hap}\;\mathrm{from monitoring}\\ +\mathrm{public awareness about}\;\mathrm{air}\;\mathrm{pollution from health burden} \end{array}$$

#### A.1.4 Expenditure

The next step is to translate this *public concern* into public *expenditure to reduce HAP*. Of all the sectors of the model, this is the one where the least amount of reliable data was available, as information on such expenditures in the past was hard to get by, and initiatives to combat *household air pollution* have so far been very rare. Therefore, we had to make certain assumptions based on expert judgment as well as calibrating to limited available data. Nevertheless, we report various tests, including sensitivity tests presented in Appendix B.1, to investigate the implications of our assumption and of potential errors in those assumptions. Here, we make our assumptions explicit.

Firstly, we obtain the growth rate in public concern, using the software's built-in *TREND*^7^ function.

$$\begin{array}{l} \mathrm{public concern growth rate}=\mathrm{TREND}\;(\mathrm{public concern about}\;\mathrm{hap},\mathrm{GROWTH TIME}\\ \mathrm{HORIZON},\mathrm{INITIAL PUBLIC CONCERN GROWTH RATE}) \end{array}$$

The smoothing (i.e. averaging) time, *growth time horizon*, is set to half a year, and the *initial public concern growth rate* is zero.

The key assumption here is that growth rate in the *expenditure to reduce household air pollution* is proportional to the growth rate in public concern. In other words, it is assumed that expenditure will be raised/reduced at a speed proportional to the speed of changes in public concern. This relationship is mediated by the constant parameter *expenditure growth rate multiplier* (equation below). This constant was estimated through calibration based on reasonable values for expenditure (according to evidence, see next page), and set at 0.75. This means that for every 1% increase in *public concern*, *expenditure* would go up by 0.75%. This *multiplier* is a crucial parameter in the model, and one which affects the model's behaviour significantly. Therefore, a sensitivity analysis is carried out on this parameter and reported on in Appendix B.1.

$$\begin{array}{l} \mathrm{change in expenditure to reduce household}\;\mathrm{air}\;\mathrm{pollution}=\mathrm{Expenditure To Reduce}\\ \mathrm{HAP}\\ {}*\mathrm{public concern growth rate}*\mathrm{EXPENDITURE GROWTH RATE MULTIPLIER} \end{array}$$

In the language of system dynamics modelling, *Expenditure to reduce HAP* is what is called a ‘stock’ variable, and the rate of *change in expenditure to reduce household air pollution* is a ‘flow’ variable. Stock variables are denoted inside a box and flow variables are shown as valves flowing into or out of stocks. The small ‘cloud’ where the flow originates signifies that the source of this change is outside the model boundary or irrelevant for our purpose.

A stock is a level that is, at any point in time, the result of the accumulation of its net flow (inflows minus outflows), plus any ‘initial’ value assigned to it. In other words, mathematically, a stock is an initial value plus the integral of its net flow. In the case of the above diagram, there is solely one inflow. Therefore:

$$\begin{array}{l} \mathrm{Expenditure to reduce household}\;\mathrm{air}\;\mathrm{pollution}=\mathrm{initial expenditure to reduce indoor}\\ \mathrm{air}\;\mathrm{pollution}+\mathrm{INTEG}\;\left(\mathrm{change in expenditure to reduce household}\;\mathrm{air}\;\mathrm{pollution}\right) \end{array}$$

The stock's *initial* value is a constant parameter for which data was not available. In 2015, total government expenditure in Nairobi for preventive and promotive health, covering TB, malaria, family planning, and environmental health has been KES 43M. If we assume that this is divided roughly equally between the four areas, each area, including environmental health, gets just under KES 11M. Korogocho is roughly 1/200 of Nairobi's population, so again if we assume that expenditure is divided evenly, Korogocho residents get roughly KES 55,000 per year. This is the total environmental health budget. Then we make the rough assumption that about a fifth of this, KES 11,000 (roughly USD 110) per year goes to fighting indoor air pollution. The population of Viwandani is 1.5 times that of Korogocho. Therefore, for Viwandani we assume a value 1.5 higher (KES 16,500). These are very low figures, in line with the information obtained in our workshops pointing out the almost complete non-attention to *household air pollution* in the government's budgeting.

Thus, we set the *initial expenditure* such that by 2015, *expenditure* goes up to the order of roughly KES 11,000 for Korgocho and KES 16,500 for Viwandani. Clearly, this is a very rough estimate that we had to make because of lack of data. However, our sensitivity analysis reveals that minor variations (±25%) in this parameter do not affect any of the policy insights obtained.

Out of all the budget allocated for HAP, according to our workshop participants, only a portion of it is effectively spent in initiatives to reduce pollution, with most of it being wasted through misallocations, in a system where corruption is still a main obstacle. To capture the effect of such misallocations, we have included a *good governance* parameter (Fig. A-7), as outlined earlier in Section 2.4.1. This is an index between zero and 1, driven for the past years by data available from the World Bank (The World Bank, 2011). The current value of 0.383 means that out of every shilling allocated to a policy or initiative, 0.383 of it is ultimately spent on that policy or initiative. The future value (green variable) is a policy variable set by the user as a target to be achieved by 2040.

The *effective expenditure* is then used in other sectors of the model to provide subsidised cook stoves, or to fund indoor air monitoring or health impact assessment (HIA) studies. The overall picture of the *Core Structure* of the model presented so far is shown below in Fig. A-8 to give an overview. Next, we will present the second (and final) model sector, the *Policy Structure*.

### A.2 Policy structure

#### A.2.1 Expenditure allocation

We assume that any funds available for fighting HAP will be divided among three types of initiatives, namely: subsidising clean cook stoves, funding indoor air monitoring, and funding HIA studies. Although in the past the little available money has been almost entirely allocated to the provision of subsidised appliances, in the future, we leave it to the user of the model to decide on this allocation. The model structure reflecting this is portrayed in Fig. A-9.

For each of the initiatives, on the left-hand side, there is one parameter in black, representing the past shares allocated to each initiative. The past setting for these shares of expenditure is 98% (virtually all of the funds) to stove subsidies, and 1% to each of the other two initiatives (virtually nothing^8^). Then, each initiative also has three green variables, which represent three step-changes in the future, which can be varied by the user. The first of these step changes happens immediately (time = 2017), while the other two take place in three-year intervals, capturing a gradual shift in policy. The equation for one of the *share* variables is shown below as an example:

$$\begin{array}{l} \mathrm{share of indoor}\;\mathrm{air}\;\mathrm{quality expenditure for monitoring}=\\ \mathrm{IF THEN ELSE}\;(\mathrm{Time}<2017,\mathrm{SHARE OF INDOOR}\;\mathrm{AIR}\;\mathrm{QUALITY EXPENDITURE}\\ \mathrm{FOR MONITORING PAST},\\ \mathrm{IF THEN ELSE}\;(\mathrm{Time}<2020,\mathrm{SHARE OF INDOOR}\;\mathrm{AIR}\;\mathrm{QUALITY EXPENDITURE}\\ \mathrm{FOR MONITORING}\;2017,\\ \mathrm{IF THEN ELSE}\;(\mathrm{Time}<2023,\mathrm{SHARE OF INDOOR}\;\mathrm{AIR}\;\mathrm{QUALITY EXPENDITURE}\\ \mathrm{FOR MONITORING}\;2020,\\ \mathrm{SHARE OF INDOOR}\;\mathrm{AIR}\;\mathrm{QUALITY EXPENDITURE FOR MONITORING}\;2023))) \end{array}$$

It is essential that the sum of the three *shares* equal one (i.e. 100%). However, the user might not adhere to this rule. Therefore, in the next step we normalise the three shares to ensure that they sum up to unity. The following is the equation for one of the *normalised* variables as an example:

$$\begin{array}{l} \mathrm{normalised share of indoor}\;\mathrm{air}\;\mathrm{quality expenditure for monitoring}=\\ \mathrm{share of indoor}\;\mathrm{air}\;\mathrm{quality expenditure for monitoring}/(\mathrm{share of indoor}\;\mathrm{air}\;\mathrm{quality}\;\mathrm{ex}‐\\ \mathrm{penditure for monitoring}+\mathrm{share of}\;\mathrm{air}\;\mathrm{quality expenditure for health impact assess}‐\\ \mathrm{ment}+\mathrm{share of}\;\mathrm{air}\;\mathrm{quality expenditure for appliance subsidies}) \end{array}$$

#### A.2.2 Indoor air monitoring and health impact assessment

Below, the structure for modelling *indoor air monitoring coverage* is shown (Fig. A-10). We have an identical structure for *health impact assessment coverage*. Therefore, we present here only one of the two structures.

*Indoor air monitoring coverage* is a stock variable capturing the extent of coverage at any point in time. This level is increased via an inflow and decreased via a natural *depreciation* outflow.

*Effective expenditure to reduce household air* pollution (blue variable on the top-left) was explained earlier. Multiplying this by the *normalized share of indoor air quality expenditure for monitoring* gives the amount of *expenditure for indoor air monitoring*. Subsequently, simply dividing this latter variable by the *unit cost of indoor air monitoring per household* gives the rate of *increase in indoor air monitoring coverage*. We need to add a MIN function to this formulation to ensure that the rate does not exceed the physical maximum. This maximum possible rate of increase is essentially the gap between *total number of households* and *indoor air monitoring coverage*, divided by the time needed to close this gap, *gap closing time constant* (assumed equal to one year). This standard structure ensures a naturally smooth asymptotic behaviour if/when we approach full coverage in the future. The complete equation for the rate of *increase* is therefore the following:

$$\begin{array}{l} \mathrm{increase in indoor}\;\mathrm{air}\;\mathrm{monitoring coverage}=\\ \mathrm{MIN}\;(\mathrm{expenditure for indoor}\;\mathrm{air}\;\mathrm{monitoring}/\mathrm{UNIT COST OF INDOOR}\;\mathrm{AIR}\;\mathrm{MONI}‐\\ \mathrm{TORING}\;\mathrm{PER}\;\mathrm{HOUSEHOLD},\\ \left(\mathrm{TOTAL number of households}-\mathrm{Indoor}\;\mathrm{Air}\;\mathrm{Monitoring Coverage}\right)/\mathrm{GAP}\;\mathrm{CLOSING}\\ \mathrm{TIME CONSTANT}) \end{array}$$

According to an appraisal made by co-authors from APHRC, we can assume that it would suffice to monitor one household out of an area comprising 1000 households. The cost of one monitoring setup is around USD 1000. Therefore, *Unit cost of indoor air monitoring* is given as roughly USD 1 (or KES 100) per household, which is the value we use in the model.

The equation for the stock of *coverage* is straightforward:

$$\begin{array}{l} \mathrm{indoor}\;\mathrm{air}\;\mathrm{monitoring coverage}=\mathrm{initial indoor}\;\mathrm{air}\;\mathrm{monitoring coverage}\\ +\mathrm{INTEG}\;(\mathrm{increase in indoor}\;\mathrm{air}\;\mathrm{monitoring coverage}-\mathrm{depreciation of investment in}\\ \mathrm{indoor}\;\mathrm{air}\;\mathrm{monitoring}) \end{array}$$

*initial indoor air monitoring coverage*, at the beginning of our simulation (year 2003), is known to be almost non-existent, and thus set to 1 household.^9^ The *depreciation* outflow is formulated as a standard first-order decay by dividing the stock level by a constant *depreciation time*, assumed to be equal to 5 years.

$$\begin{array}{l} \mathrm{depreciation of investment in indoor}\;\mathrm{air}\;\mathrm{monitoring}=\\ \mathrm{Indoor}\;\mathrm{Air}\;\mathrm{Monitoring Coverage}/\mathrm{INVESTMENT DEPRECIATION TIME} \end{array}$$

The stock of *coverage* is then divided by the *total number of households* to obtain the *proportion of households covered by indoor air monitoring* indicator. We used this variable earlier to formulate the level of *public awareness* about air pollution.

#### A.2.3 Access to clean stoves

Next, we focus on the indicator *number of households using clean stoves* (Fig. A-11), which we used earlier to obtain the *prevalence of clean stoves*.

The *number of households using clean stoves* (indicated in red) is a function of *number of house owning clean stoves*, as well as *fuel cost of LPG stove relative to kerosene stove*. The premise here is that as the cost of using a clean LPG stoves rises relative to the use of a kerosene stove, fewer people, even among those who already own a gas stove, would be using clean LPG stoves. This is captured in the *effect of relative fuel prices on clean stoves usage* graph function (Fig. A-12), which was estimated in consulting with local experts:

This graphical function postulates that as long as an LPG stove is equally or less expensive to use than a kerosene stove, households owning an LPG stove will use it 85% of the time (see the first point on the graph at (1, 0.85)). The 85% estimate is to account for ‘fuel stacking’, which is the use of multiple fuels/stoves at one time. As LPG becomes relatively more expensive, the percentage of the time that households owning clean stoves would actually use them drops quickly. In the model, as shown in the graph, we assume that this percentage would reach zero gradually by the point where using an LPG stove is 10 times more expensive than a kerosene stove (see the (10, 0) point). Therefore:

$$\begin{array}{l} \mathrm{number of households using clean stoves}=\mathrm{Number Of Households Owning Clean}\\ \mathrm{Stoves}*\\ \mathrm{EFFECT OF RELATIVE FUEL PRICES}\;\mathrm{ON}\;\mathrm{CLEAN STOVE USAGE}\;(\mathrm{fuel cost of}\;\mathrm{lpg}\\ \mathrm{stove relative to kerosene stove}) \end{array}$$

Later in this section, we will see how the *fuel cost of LPG stove relative to kerosene stove* variable is formulated. But first, we will focus on the stock of *number of households owning clean stoves*. The initial value for this stock, *initial number of households using clean* stoves, is given by the NUHDSS dataset. Changes in this stock are a result of households acquiring clean cook stoves, either subsidised or on the free market. An upper limit structure reflecting what is physically possible is included in the same way as for the stock of *indoor air monitoring coverage*, as seen earlier. The equations for the stock and its inflow are therefore:

$$\begin{array}{l} \mathrm{number of households owning clean stoves}=\mathrm{initial number of households owning}\\ \mathrm{clean stoves}+\mathrm{INTEG}\;\left(\mathrm{change in households with access to clean stoves}\right)\\ \mathrm{change in households with access to clean stoves}=\\ \mathrm{MIN}\;(\mathrm{households acquiring market price clean stoves}+\mathrm{households acquiring subsi}‐\\ \mathrm{dised clean stoves},(\mathrm{TOTAL number of households}-\mathrm{Number Of Households Owning}\\ \mathrm{Clean Stoves})/\mathrm{GAP}\;\mathrm{CLOSING TIME CONSTANT}) \end{array}$$

Later in this section we will see how *households acquiring market price clean stoves* is formulated. But first, the number of subsidised stoves distributed equals to the funds allocated divided by the amount of subsidy per stove. The money available is obtained by multiplying the *effective expenditure to reduce household air pollution* by *normalised share of air quality expenditure for appliance subsidies*. The cost of each subsidised stove for the government equals its price multiplied by the *proportion of appliance prices subsidised*. This proportion is set to 0.5, estimated by co-authors from APHRC. The *Price of clean stoves* is set according to data in the past (KES 2000),^10^ and the *target* future price is decided upon by the user as a policy variable.

$$\begin{array}{l} \mathrm{households acquiring subsidised clean stoves}=\mathrm{expenditure for clean stove subsidies}/\\ \left(\mathrm{price of clean stoves}*\mathrm{PROPORTION OF APPLIANCE PRICES SUBSIDISED}\right) \end{array}$$

As for the rate of acquisition of clean cook stoves on the market, the Fig. A-13 demonstrates that in our model, we have assumed three drivers for it: stove prices, relative fuel prices, as well as public concern for HAP. *Relative* variables are values normalized via dividing by *initial* values. The strength of each one of the above drivers is captured using an elasticity formulation, making percentage changes in the acquisition rate proportional to percentage changes in its drivers. The elasticities are based on the literature where available, and otherwise estimated numerically (using Monte Carlo methods) to give the best possible fit with the available time-series data for *number of households owning clean stoves*. The *initial* acquisition rate is also estimated in the same way. The final equation for the acquisition rate is^11^:

$$\begin{array}{l} \mathrm{households acquiring market price clean stoves}=\\ \mathrm{INITIAL HOUSEHOLDS ACQUIRING MARKET PRICE CLEAN STOVES}\\ {}*\mathrm{relative fuel cost of}\;\mathrm{lpg}\;\mathrm{stove relative to kerosene stove}\;ˆ\;\mathrm{ELASTICITY OF CLEAN}\\ \mathrm{STOVE ACQUISITION TO FUEL PRICES}\\ {}*\mathrm{relative price of clean stoves}\;ˆ\;\mathrm{ELASTICITY OF CLEAN STOVE ACQUISITION TO}\\ \mathrm{STOVE PRICES}\\ {}*\mathrm{relative public concern about}\;\mathrm{hap}\;ˆ\;\mathrm{ELASTICITY OF CLEAN STOVE}\\ \mathrm{ACQUISITION TO PUBLIC CONCERN} \end{array}$$

Earlier, we mentioned *fuel cost of LPG stove relative to kerosene stove* as a driver of clean stove usage. Fig. A-14 depicts how this driver is formulated:

The *relative cost* is calculated by dividing *monthly fuel cost of an LPG stove* by *monthly fuel cost of a kerosene stove*. Each of the *costs* is obtained by multiplying the *price* of the fuel by its *monthly usage*. Data for *Monthly usage* was obtained based on focus groups with slum residents. Residents said that each 6 kg LPG canister lasts them about 3 months. As for kerosene, residents said that they used about KES 50 per day, which translates to 24 l per month, at a current price of KES 64 per litre. Past *price* data comes from the Kenyan National Bureau of Statistics (KNBS). Future *prices* are policy variables (in green) which the user can decide to increase/decrease in three steps separated by three years each.

It should be noted that in our conversations with residents of Korogocho and Viwandani, we found out that the bottleneck for using LPG stoves for many families is the high cost of LPG refill cylinders which are usually offered in 6 or 13 kg, rather than the cost of the stove itself. This bottleneck is not included in the model. We expect, however, that the associated dynamics be of a similar nature to when we assume the bottleneck to be the stove itself.

#### A.2.4 Access to clean lighting

Finally, we will explore how the *number of households using clean lighting* is formulated (Fig. A-15). This indicator (highlighted in red) is assumed to be equal to the *number of households owning clean lighting* stock variable, i.e. all households owning clean lighting use that type of lighting all the time. The stock of *number of households owning clean lighting* is initialized using available data from NUHDSS. The rate of *change* in the stock is driven by two factors: *electricity coverage* and *price of clean lighting*. *Electricity coverage* data was made available via NUHDSS. *Target electricity coverage 2040* is set at 100%, assuming that by 2040 there will be full coverage in our two slums of interest. From this *coverage* data, *electricity coverage growth* rate is obtained by subtracting *electricity coverage delayed* from *electricity coverage* and dividing it by *growth rate time horizon* (=0.5 year). *Electricity coverage delayed* is simply a fixed delay of *electricity coverage*, the delay time being set equal to *growth rate time horizon*.^12^

$$\begin{array}{l} \mathrm{electricity coverage growth}=\\ \left(\mathrm{electricity coverage}-\mathrm{Electricity Coverage Delayed}\right)/\mathrm{GROWTH TIME HORI}‐\\ \mathrm{ZON}\\ \mathrm{Electricity Coverage Delayed}=\mathrm{DELAY FIXED}\;(\mathrm{electricity coverage},\\ \mathrm{GROWTH TIME HORIZON},\mathrm{ELECTRICITY COVERAGE}\;2002) \end{array}$$

Subsequently, the *indicated change in households with access to electricity* is the *total number of households* multiplied by *electricity coverage growth*, which assumes that the rates of growth in electricity coverage is almost equal to the rate of growth in the *number of households owning clean lighting*. The only difference is that this latter is subsequently modified by an effect from the *price of clean lighting*. Past *price* data was provided by local APHRC experts, and future *target price* is set by the user as a policy variable (in green), to be reached linearly by 2040. *Relative price of clean lighting* is the normalized version obtained by dividing current *price* by its initial *value*. The effect is then formulated using an elasticity formulation that is multiplied by *indicated change*. The *elasticity of clean lighting acquisition to lighting prices* is set at −0.6 (Fouquet and Pearson, 2012, p. 17).

$$\begin{array}{l} \mathrm{change in households with access to clean lighting}=\mathrm{indicated change in}\\ \mathrm{households with access to electricity}\\ {}*\mathrm{relative price of clean lighting}\;ˆ\;\mathrm{ELASTICITY OF CLEAN LIGHTING}\\ \mathrm{ACQUISITION TO LIGHTING PRICES} \end{array}$$

Like our previous flow variables, a cap is applied to this rate to reflect a physical maximum (i.e. the second argument of the MIN function). This auxiliary part of the equation is not shown here for the sake of simplicity.

Putting together all the pieces of structure described in this section gives a complete picture of the model's *Policy Structure* (Fig. A-16).

## Appendix B Sensitivity testing

In this section, we will report several Monte Carlo tests to investigate the sensitivity of the model's behaviour to some of the parameters. In such tests, we specify an uncertainty range and stochastic distribution for the parameter(s) under investigation, and based on this configuration, the software will run a large number of simulations, which we set to 300 times, while randomly varying the specified parameter according to the specified range and distribution function. Subsequently, we can visualise the results as sensitivity graphs with confidence bounds. The two parameters chosen for sensitivity testing here are ones that were deemed to be particularly uncertain and influential on model behaviour in the course of building the model. There are of course other uncertain parameters as well, but these were considered not as uncertain or crucial in terms of behaviour as the two parameters reported here.

### B.1 Expenditure growth rate multiplier

First, we examine the sensitivity of model behaviour to the *expenditure growth rate multiplier*, a parameter that determines the strength of the relationship between changes in *public concern about household air pollution* on the one hand, and *expenditure to reduce household air pollution* on the other. In our model, this parameter is set equal to 0.75, which means that if the public becomes 10% more concerned about household air pollution, expenditure to reduce household air pollution will go up by 7.5%. However, this is only an assumption, and in the real world the strength of this relationship might be lower or higher than that. Therefore, we run a Monte Carlo analysis, with the assumptions of *Scenario II*, where we vary this parameter between 0.25 and 1 based on a uniform distribution. The result of this test on the variable *expenditure to reduce HAP* are visualised in Fig. B-1.

In 50% of simulations, results lie in the yellow region, in 75% of simulations in the green region, in 95% within the blue region, and in every case within the grey region. This test demonstrates that model behaviour is highly sensitive to changes in this uncertain parameter. Also, the model is built such that expenditure initially rises exponentially as the public becomes more concerned, and more money goes into health impact assessment and monitoring. Afterwards, the spending reaches a plateau where HAP has not yet reached acceptable levels, but at this point that is entirely attributable of outdoor air pollution, which is assumed to stay constant in the model. In the real world, under such circumstances, expenditure would be quickly diverted from an exclusive focus on HAP once almost all households own clean appliances, and thus the expenditure graph should go down rather than stay constant. However, such structure for limiting expenditure is not included in our model as the circumstances where it would be needed are not likely to occur within our normal range of parameters.

This sensitivity test reveals the significant sensitivity of our model's behaviour to the one uncertain parameter which characterises how strongly changes in public concern translate into changes in expenditure. In more intuitive terms, if increases in public concern do not lead to any substantial increase in expenditure, then the reinforcing loops described earlier would not be set in motion to bring any visible improvements in household air quality. Thus, empirical research aimed at quantifying the strength of this relationship is needed to build more confidence in the model.

### B.2 Public concern delay

For this second test, we consider the *public concern delay* parameter because it determines how long it takes on average for indoor air monitoring and indoor-air-related health impact assessment studies to generate concern within the public. Such information delays of the system give it its dynamic behaviour and are therefore crucial in a long-term policy analysis model. Having assumed an uncertain value of two years for this parameter, it is important to investigate how potential errors in this estimate will affect our results.

Thus, we run a Monte Carlo analysis with *public concern delay* as our uncertain parameter, with 300 randomly generated inputs with a normal distribution (mean: 2 years, standard deviation: 0.4 years, range: 0.5 to 3.5 years), once again together with our assumptions for *Scenario II*. Fig. B-2 plots the results of this configuration for *household air pollution*. The solid dark green curve in the middle is *Scenario II* itself. It can be observed that the model is sensitive to this parameter. The shorter the delay, the more quickly studies generate concern and concern brings in available funds, and the steeper the reduction in *household air pollution*. Also notable is that in all cases air quality eventually reaches a plateau with no further improvements to be expected from within the dwellings, the rest of the pollution coming entirely from the outdoors.

One implication of this analysis is that the sooner the results of health impact assessment and monitoring studies are brought to the fore of the public's attention, the more quickly our self-reinforcing mechanisms would be put in motion, with the potential to achieve full prevalence of clean cook stoves several years earlier (Fig. B-3). Note that even in the most rapid take-up scenarios (shortest delays) we never reach 100% prevalence, as, according to our field research, even when all households own an LPG cook stove, they do not always use it, a practice known as *fuel* stacking: For certain foods, households would use traditional cook stoves, even when they have access to clean LPG stoves.

### B.3 Bivariate sensitivity

In this section, we run a Monte Carlo sensitivity test where the above two parameters are allowed to vary in tandem. The result, as shown in Fig. B-4, show considerably wider confidence bounds, especially in the 50% bounds towards higher pollutant concentrations. This tells us that, according to the model, simultaneous random variations in both parameters are more likely to lead to less pronounced improvements in household air pollution. In other words, the test reveals that, given this uncertainty in parameters, our model is more likely to overestimate, rather than underestimate, improvements in household air pollution.

## Appendix D List of all parameters

This table excludes policy/scenario variables for various scenarios, which are reported separately in Appendix C above.

## Appendix E Supplementary data

Korogocho base model, Vensim file Image 1

Korogocho Scenario I model, Vensim file Image 2

Korogocho Scenario II model, Vensim file Image 3

Korogocho Scenario III model, Vensim file Image 4

Korogocho datasheet, Excel file Image 5

Viwandani base model, Vensim file Image 6

Viwandani Scenario Imodel, Vensim file Image 7

Viwandani Scenario II model, Vensim file Image 8

Viwandani datasheet, Excel file Image 9

Sensitivity run for 'expenditure growth multiplier', Vensim file Image 10

Sensitivity run for 'public concern delay', Vensim file Image 11

## Footnotes

1 For a full list of parameter differences, see Appendix D.

2 Kenya is working to achieve ‘universal access’ to electricity by 2020, whereby 95% of homes will be covered, through the Last Mile Connectivity Project (Kenya Power, n.d.). However, USAID predicts that by 2020, 70–80% of the population will have access (USAID, 2016). With this in mind, in our model we assumed that we reach ‘universal access’ to electricity in the slums by 2040, the end of our simulation period.

3 In reality, outcomes may not necessarily be this bleak, because there are myriad other potential self-reinforcing mechanisms not included in our model that might change things as we move towards 2040, such as for instance effects of breakthrough technological advances, improvements in income and quality of life, or major slum upgrade programmes that may fundamentally change the structure of the system.
